# Nationwide Molecular Surveillance of Pandemic H1N1 Influenza A Virus Genomes: Canada, 2009

**DOI:** 10.1371/journal.pone.0016087

**Published:** 2011-01-07

**Authors:** Morag Graham, Binhua Liang, Gary Van Domselaar, Nathalie Bastien, Carole Beaudoin, Shaun Tyler, Brynn Kaplen, Erika Landry, the National Influenza A/H1N1pdm Genomics Study Team (NIGST), Yan Li

**Affiliations:** 1 National Microbiology Laboratory, Public Health Agency of Canada, Winnipeg, Manitoba, Canada; 2 Department of Medical Microbiology, University of Manitoba, Winnipeg, Manitoba, Canada; 3 Department of Computer Science, University of Manitoba, Winnipeg, Manitoba, Canada; 4 Department of Community Health Sciences, University of Manitoba, Winnipeg, Manitoba, Canada; The University of Hong Kong, Hong Kong

## Abstract

**Background:**

In April 2009, a novel triple-reassortant swine influenza A H1N1 virus (“A/H1N1pdm”; also known as SOIV) was detected and spread globally as the first influenza pandemic of the 21^st^ century. Sequencing has since been conducted at an unprecedented rate globally in order to monitor the diversification of this emergent virus and to track mutations that may affect virus behavior.

**Methodology/Principal Findings:**

By Sanger sequencing, we determined consensus whole-genome sequences for A/H1N1pdm viruses sampled nationwide in Canada over 33 weeks during the 2009 first and second pandemic waves. A total of 235 virus genomes sampled from unique subjects were analyzed, providing insight into the temporal and spatial trajectory of A/H1N1pdm lineages within Canada. Three clades (2, 3, and 7) were identifiable within the first two weeks of A/H1N1pdm appearance, with clades 5 and 6 appearing thereafter; further diversification was not apparent. Only two viral sites displayed evidence of adaptive evolution, located in hemagglutinin (HA) corresponding to D222 in the HA receptor-binding site, and to E374 at HA2-subunit position 47. Among the Canadian sampled viruses, we observed notable genetic diversity (1.47×10^−3^ amino acid substitutions per site) in the gene encoding PB1, particularly within the viral genomic RNA (vRNA)-binding domain (residues 493–757). This genome data set supports the conclusion that A/H1N1pdm is evolving but not excessively relative to other H1N1 influenza A viruses. Entropy analysis was used to investigate whether any mutated A/H1N1pdm protein residues were associated with infection severity; however no virus genotypes were observed to trend with infection severity. One virus that harboured heterozygote coding mutations, including PB2 D567D/G, was attributed to a severe and potentially mixed infection; yet the functional significance of this PB2 mutation remains unknown.

**Conclusions/Significance:**

These findings contribute to enhanced understanding of Influenza A/H1N1pdm viral dynamics.

## Introduction

Influenza viruses (family *Orthomyxoviridae*) possess a segmented, negative polarity, single-stranded RNA genome and lipid-enveloped virions [1]. Genetic diversity in influenza virus results from a high replication error rate associated with low-fidelity RNA polymerase, and the reshuffling (or reassortment) of gene segments among co-infecting strains [1]–[3]. These genetic shifts periodically lead to antigenically novel strains that emerge as pandemic viruses [1]. In April 2009, a novel triple-reassortant swine influenza A/H1N1 virus (also known as SOIV) was detected, with human cases first identified in Mexico and California [4]; thereafter, the pandemic 2009 influenza A (H1N1) virus (designated hereafter as “A/H1N1pdm”) spread globally as the first influenza pandemic of the 21^st^ century. During the 2009–2010 pandemic, A/H1N1pdm became the dominant circulating virus. Efficient human-to-human transmission was observed, with clinical attack rates of 20–50% [5]. Laboratory-confirmed A/H1N1pdm cases occurred in over 214 countries, with over 18,114 worldwide deaths attributed as of 23 May 2010 [6].

The 13.5-kb influenza A virus genome is comprised of eight segments coding for up to 11 viral proteins: three proteins encoding the RNA-dependent RNA polymerase complex (PA, PB1, and PB2); nucleoprotein (NP); nonstructural (NS1 and NEP (formerly known as NS2)) and matrix (M1 and M2) proteins, and two glycoproteins, hemagglutinin (HA) and neuraminidase (NA) [1]. The pro-apoptotic but nonstructural polymerase basic 1 frame 2 (PB1-F2) protein encoded by segment 2 terminates prematurely in A/H1N1pdm viruses [7].

The Canadian provinces of Quebec (PQ) and Ontario (ON) first identified cases of influenza-like illness (ILI) with travel histories or exposure to travelers returning from Mexico within 2 weeks of outbreak detection (weeks 03–04 relative to the global outbreak initiated April 1 2009). Thereafter, A/H1N1pdm was detected in British Columbia (BC), Alberta (AB) and Nova Scotia (NS) [8] and then spread across Canada in two distinct waves. According to Canada's national sentinel surveillance system (FluWatch), during wave 1 spanning global outbreak weeks 03 to 22 (April 1 to August 29 2009, representing FluWatch weeks 15 to 34) 6676 laboratory-confirmed cases were attributed to A/H1N1pdm. As of June 19 2010, 33,521 laboratory-confirmed cases had been attributed to A/H1N1pdm during wave 2 (commencing August 30 2009). Hence, a sum total of 40,197 A/H1N1pdm cases were reported to the Public Health Agency of Canada (PHAC). However, as neither confirmation nor reporting of clinically diagnosed cases was required, this figure significantly underestimates the true extent of A/H1N1pdm encountered in Canada. As elsewhere, the vast majority of A/H1N1pdm-infected Canadians experienced predominantly mild uncomplicated, self-limiting, upper respiratory tract illness; fewer than 20% of cases required hospitalization (Source: FluWatch).

The A/H1N1pdm virus continues to circulate worldwide, giving rise to concerns about genotypic diversity that might evolve to enhance virus transmissibility and/or pathogenicity and affect vaccine efficacy. An earlier study noted early diversification of the A/H1N1pdm virus into seven distinct lineages, designated as clades 1–7, with spatial patterning within 19 weeks of the pandemic's initiation [9]. Herein, we report whole-genome investigations for A/H1N1pdm viruses sampled nationwide over a broader timeframe of 33 weeks (April 16 2009 - December 5 2009) during the 2009 first and second waves in Canada; representing *n* = 178 and 57 unique virus genomes, respectively. We then conducted analyses to gain insight into 1) how A/H1N1pdm influenza spread spatially and temporally during the nationwide epidemic, and 2) whether mutated A/H1N1pdm protein residues influenced infection severity. Understanding the spatial and temporal spread of A/H1N1pdm lineages in Canada may provide insights into disease trajectory for future influenza pandemic planning. Determining any correlation between mutated protein residues and infection severity is important for vaccine development, and could be important for resource allocation should identified mutations arise in at risk populations.

## Results

### Multiple introductions of A/H1N1pdm into Canada, generating complex spatial patterns

**Figure 1:**
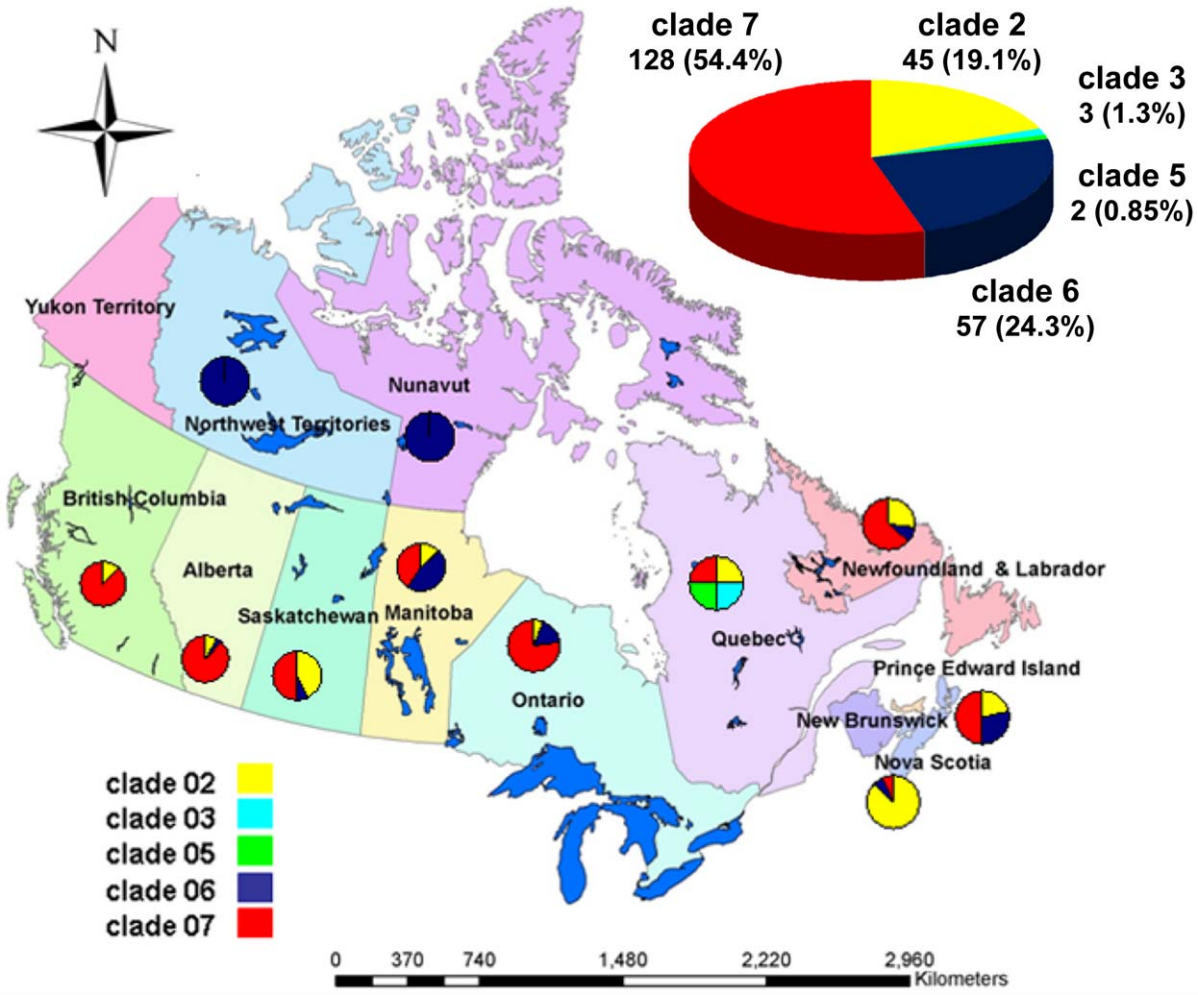
Geographical and clade distributions for A/H1N1pdm viruses sampled in Canada. Phylogenetic analysis was performed with two hundred thirty five viruses sampled in Canada and linked to their corresponding clades, as shown in Figure 2.

**Figure 2:**
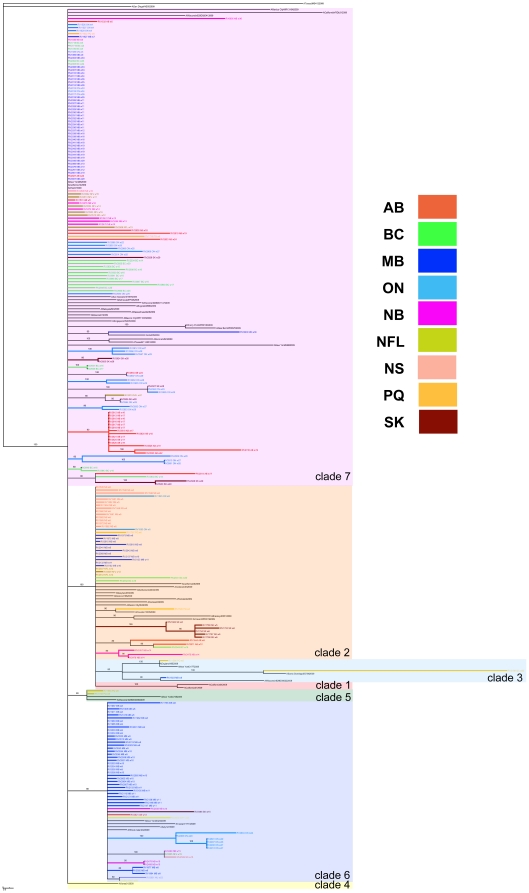
Spatial patterning of Canadian A/H1N1pdm viruses. Phylogenetic trees were inferred using the neighbour-joining distance method, with genetic distances calculated by maximum likelihood using Kimura's two-parameter model (K2P) in MEGA 4.0. The resultant consensus tree was generated using the Summarization of Split Support on Phylogenetic Trees (SumTrees) program ver. 1.02. Lineages are colour-coded according to the region of virus collection.

**Figure 3:**
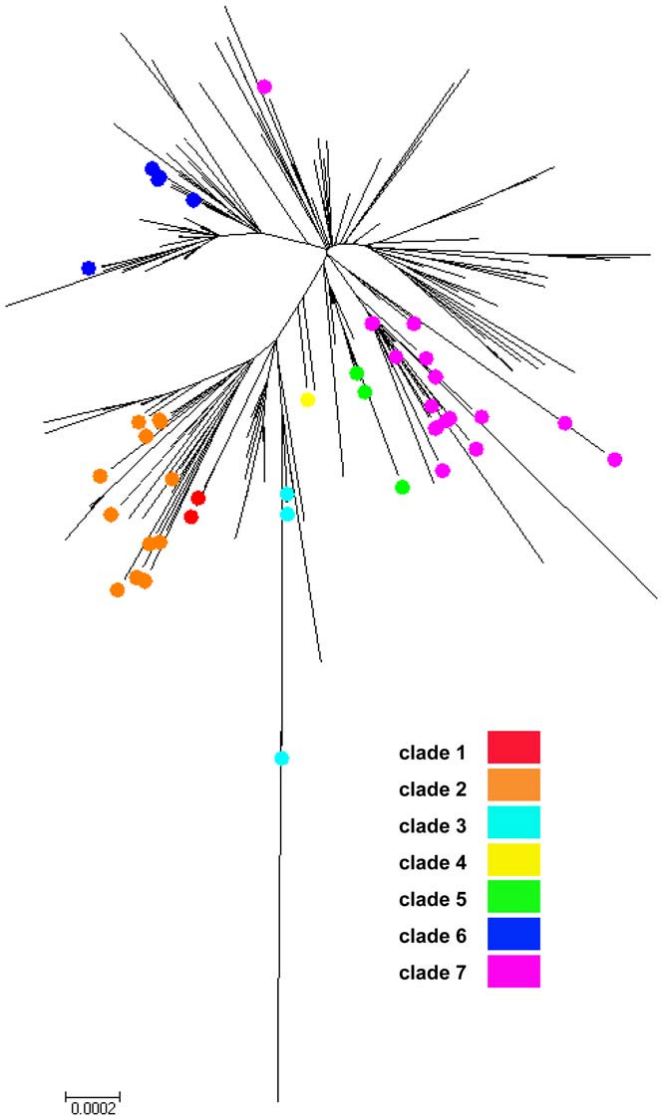
Phylogenetic relationships for A/H1N1pdm viruses sampled from Canada and globally. Phylogenetic tree inferred for two hundred thirty five virus whole genomes sampled from Canada and representative global reference genomes using the Neighbour-joining (NJ) method. Scale bar indicates the number of nucleotide substitutions per site. Colour-coding: clade 1 (not shown); clade 2 (orange); clade 3 (teal); clade 4 (yellow); clade 5 (green); clade 6 (blue), and clade 7 (pink).

Supplemented with 1198 whole-genome sequences available globally as of June 2 2010, the concatenated A/H1N1pdm sequences sampled from Canadian provinces and territories (*n* = 235) were subjected to phylogenetic analysis. Our phylogenetic inference supports that multiple A/H1N1pdm lineages were introduced into Canada generating complex spatial patterns (Figures 1, 2 and 3). Presence in all Canadian sequences of non-clade 1 mutations (namely HA (P83S [P100S], and I321V [I338V]) and PA (P224S)) agrees with prior reports of limited geographic dissemination of clade 1 isolates [9].

The first Canadian specimen (RV1595/09 (PQ) representing a mild case acquired April 16 2009 or week 03 of the global outbreak) belonged to clade 2 (Tables S1 and S2). Characterized by two amino acid substitutions relative to ancestor sequences: PA (M581L) and NP (T373I), clade 2 viruses quickly disseminated worldwide [9], and nationwide (this study). In a recent non-phylogenetic, graph-based genealogical analysis (*SeqTrack*) based on 433 HA and NA global outbreak sequences, RV1595/09 exhibited inferred ancestry dating to March 30 2009 [10], a timeframe also shared with the first globally sampled and sequenced A/H1N1pdm virus (clade 1; A/California/04/2009) and 11 other viruses, including another clade 2 virus from Canada (designated RV1644/09 from Alberta (AB) associated with a severe infection). Clade 2 viruses represented 19.1% (45/235) of the sampled Canadian sequences (Figure 1). However, 13 (29%) were sampled during global outbreak week 05, representing the first identified (and most characterized cluster) of human influenza A/H1N1(2009) (H1N1pdm) infections in Nova Scotia (NS), Canada. The NS genomes data set confirms prior epidemiological data stating that all cases (all mild) had contact with a return traveler from Mexico and transmission occurred through human-to-human transmission from a single index case [8]. Clade 2 viruses appeared shortly thereafter in Ontario (ON) and Saskatchewan (SK). A previous report that no isolates collected globally were members of clade 2 following outbreak week 08 [9] is not supported by our findings, since clade 2 viruses were present (albeit in lower prevalence) throughout wave 1, and even as late as outbreak week 30 during Canada's wave 2 (Table S2).

Clade 3 (represented by A/Arizona/01/2009) was detected globally several weeks after clades 1 and 2; although no fixed amino acid changes are common to all clade 3 isolates [9]. Clade 3 first appeared in Canada (represented by RV1586/09 - PQ) during week 04 with inferred ancestry in the previous week [10]. However as noted elsewhere, clade 3 also did not persist in Canada; a sum total of only three clade 3 viruses were identified amongst Canadian viruses in PQ (2) and Manitoba (MB) (1) regions (Figures 1 and 3; Table S2). Interestingly, the last clade 3 virus (RV3189/09 - PQ) was not sampled until outbreak week 32.

Clade 5, characterized by amino acid changes in the NP (V100I) and NA (V106I and N248D) was only observed twice in Canada (RV1758/09 and RV1954/09) in PQ during outbreak weeks 06 and 08, respectively (Figures 1 and 3; Table S2) with inferred ancestries in the same week 06 [10].

Clade 6, characterized by two non-epitope, unique amino acid substitutions in HA (K-15E [K2E] and Q293H [Q310H]), appeared later in Canada starting in week 06 (RV1765/09 - MB). Clade 6 viruses accounted for 24.3% (57/235) of the sampled Canadian viruses. Amongst this study's sequenced data set, clade 6 viruses dominated in MB (*n* = 40) up to outbreak week 11. Sequenced clade 6 viruses also were detected in 8 other regions (AB, New Brunswick (NB), Newfoundland and Labrador (NFL), NS, Nunavut (NU), Northwest Territories (NWT), ON, and SK), but were fewer (range: 1–6) (Figures 1 and 3; Table S2).

Clade 7 originated late in March 2009 and appeared in New York a few weeks after clades 1 and 2 were isolated in Mexico and California [9]. Clade 7 sequences, characterized by fixed amino acid changes in the NP (V100I) and NA (V106I and N248D) that are also found in clades 5 and 6 as well as by unique amino acid changes in mature HA (S203T) and NS1 (I123V), first appeared in Canada in ON (RV1526/09, RV1527/09, and RV1529/09) during global outbreak week 04 (Table S3). RV1527/09 exhibited inferred ancestry in the same week [10]. Clade 7, globally the most widely circulating clade, also represented the most heterogeneous cluster and the largest fraction - 54.5% (128/235) - of the sampled Canadian sequences (Figure 2). Clade 7 viruses were spatially distributed across 9 sampled regions (AB, British Columbia (BC), MB, NB, NFL, NS, ON, PQ, and SK) (Figure 1; Table S2).

**Figure 4:**
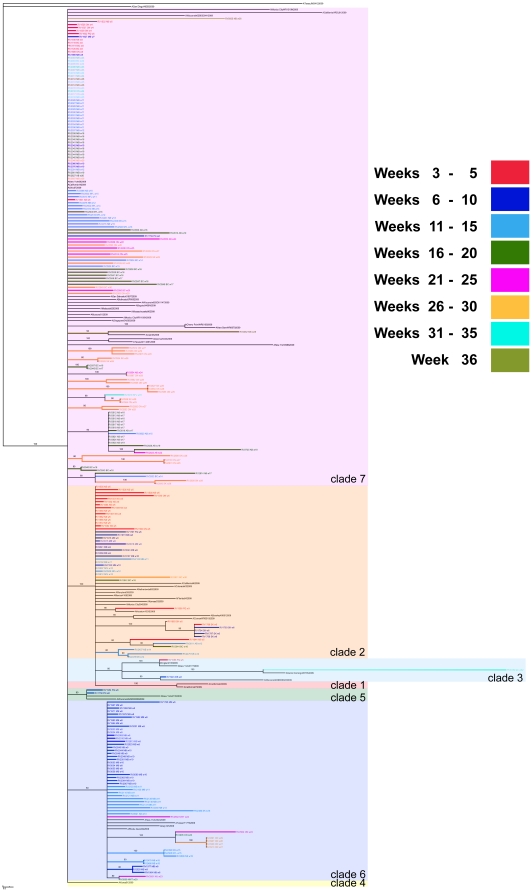
Temporal patterning of Canadian A/H1N1pdm viruses. Phylogenetic trees were inferred using the neighbour-joining distance method, with genetic distances calculated by maximum likelihood using Kimura's two-parameter model (K2P) in MEGA 4.0. The resultant consensus tree was generated using the Summarization of Split Support on Phylogenetic Trees (SumTrees) program ver. 1.02. Clade distributions are colour-coded according to 1, 3 or 5 week time periods corresponding to virus collection dates.

This study's whole-genome data set provides high density sampling of viruses circulating in Canada throughout weeks 05 through 19 of the global A/H1N1pdm wave 1 outbreak (*n* = 168) and additional sampling of circulating A/H1N1pdm viruses during weeks 23 through 31 (*n* = 42) (Figure S1), which just precedes Canada's wave 2 peak (occurring at outbreak week 32; Figure S2). The distributions of A/H1N1pdm virus clades in Canada are depicted in Figures 2, 3, and 4. Three clades (2, 3, and 7) were identifiable within the first two weeks of A/H1N1pdm appearance in Canada, with clades 5 and 6 appearing two weeks thereafter. Thus, multiple introductions of phylogenetically-distinct A/H1N1pdm occurred into Canada from the Americas. Further diversification in Canada beyond these five previously described clades is not apparent within the 33 week sampling period (Figure 3). Overall, there was limited association between virus clades and their originating geographic regions (Figures 1 and 2), indicating multiple introductions had occurred. No association existed between the phylogenetic positioning of the viruses and their sampling collection week (Figure 4).

### Limited A/H1N1pdm genetic diversity during temporal and spatial spread in Canada

Phylogenetic analysis of the 235 unique whole-genomes for Canadian A/H1N1pdm viruses sampled between April 16 and December 5 2009 revealed polymorphic sites in all eight segments of the viral genomes (Table 1). Coding variations are summarized in the Supporting Information (Table S2). A sum total of 1124 synonymous (s) and 917 non-synonymous (ns) variants were observed across the data set, corresponding to genome mean of approximately 7.82×10^−4^ nucleotide changes per gene site and 9.00×10^−4^ coding substitutions per protein site. Coding variants were non-uniformly distributed across the segments: HA (352); NA (178); PB1 (172); NP (127); PB2 (32); PA (28); NS1 (18); M1 (4); M2 (4), and NS2/NEP (2) (Table S2). However, A/H1N1pdm clade-specifying variations (relative to ancestor sequences) accounted for 68% (622/917) of the ns variants. Overall these findings from Canada support that A/H1N1pdm virus is evolving, but not excessively relative to other H1N1 influenza A viruses.

**Table 1:** Genetic diversity of A/H1N1pdm coding sequences sampled in Canada.

Viral Segment	Gene	NT^a^ Substitutions per Gene Site	AA^b^ Substitutions per Protein Site
1	PB2	2.14×10^−4^	2.32×10^−4^
**2**	**PB1**	**1.64×10^−3^**	**1.47×10^−3^**
3	PA	2.09×10^−4^	2.87×10^−4^
**4**	**HA**	**1.56×10^−3^**	**1.98×10^−3^**
**5**	**NP**	**1.65×10^−3^**	**8.83×10^−4^**
**6**	**NA**	**1.67×10^−3^**	**2.36×10^−3^**
7	M1	4.15×10^−4^	9.44×10^−5^
7	M2	1.17×10^−4^	1.74×10^−4^
8	NS1	2.53×10^−4^	6.19×10^−4^
8	NEP (NS2)	9.32×10^−5^	1.31×10^−4^

a NT: nucleotide; in viruses sampled from 235 unique subjects;

b AA: amino acid; in viruses sampled from 235 unique subjects;

Viral segments exhibiting highest genetic diversity are highlighted in bold font.

Aside from expected diversity for the antigenic viral glycoproteins (HA and NA), high ns/s (coding/non-coding) ratios also were observed in the genes encoding nucleoprotein (NP) and polymerase basic protein 1 (PB1) (Table S2). PB1 diversity in the Canadian sampled A/H1N1pdm viruses was somewhat surprising given a paucity of PB1 adaptive mutations, relative to PB2 and PA variations, described in the literature.

In order to track the antigenic evolution of A/H1N1pdm, we investigated potential antigenic drift in HA. Amino acid substitutions were observed in a limited number (9) of the Canadian viruses at characterized hemagglutinin antigenic sites: Ca (7) - R205K in Ca1 (6) and P137S Ca2 (1); S74N in Cb (1); and A186T in Sb (1) (Table S2) [11], [12]. Herein the amino acid residues represent the H1 numbering scheme exclusive of the signal peptide. These specimens were collected in AB, BC, MB, and ON during both waves; all were isolated from known mild or unknown cases. All six of the R205K Ca1 variants were clade 6 viruses isolated in southwestern ON during wave 2 (weeks 20, 24, 26, 27 and 29); thus, could have emerged from a regional single point introduction.

Glycosylation of HA plays a role in antigenic variation by masking antigenic sites, conveying resistance to neutralizing antibodies [13]. Few glycosylation variants were noted in the Canadian sampled viruses, and most generated loss of *N*-glycosylation sequons (N87K; RV1532/09; residue 104 in propeptide) or loss of potential “*Cand1*” glycosylation sites (N56D; N228D; Y230H in the HA1 globular head, and a mixed site N473N/R in HA2 (RV2810/09; residue 490 in propeptide) [11]. All of these variants were attributed to mild or unknown influenza cases. Thus, additional *N*-glycosylation sequons have not been acquired by A/H1N1pdm viruses since introduction in Canada.

Only six coding substitutions were observed within the HA receptor-binding site (RBS): one A186T variant in the 190-helix (184–191); and 5 residue 222 variants (D222E and D222G) in the 220-loop (mature peptide residues 218–225). Residue 222 (225 in H3 numbering), has garnered attention because it plays a role in conferring specificity to α2–6 sialylated glycans present in the human upper airway, and may modulate binding to the human lower respiratory tract pneumocytes expressing α2–3 sialylated glycans [14]. However, these five Canadian HA D222 variant viruses were not geographically clustered, did not spread, and the status of the associated infections remains unknown.

A sporadic HA variant of note, E374K (residue 391 in HA propeptide), was observed in 8 Canadian viruses from 5 Canadian regions spanning outbreak weeks 17 to 36. In the available HA crystal structure, residue 374 (corresponding to HA2-subunit position 47) is directed into the cavity where the fusion peptide resides in the mature fusion-ready form of the HA molecule [12], [15]. Hence, this mutation may alter salt bridge patterns and stability in a region of the HA oligomerization interface that is important for membrane fusion. Residue 374 is also near to a region identified in HA-neutralizing antibody structures that target a known antigenic site in the HA stem region [16]. These eight HA E374K viruses were associated with mild infection (1) or cases of unknown severity (7).

NA residue positions 118, 119, 151, 152, 156, 179, 180, 223, 225, 228, 247, 277, 278, 293, 295, 368, and 402 are important for contacting sialoligosaccharide substrates directly; participating in catalysis, or for providing a structural framework [17]. Substitutions within these sites were observed in only 2 Canadian A/H1N1pdm viruses, namely NA E119K (RV2984/09 sampled in global outbreak week 29) and N295S (1: RV0005-10/2009 sampled in week 36). We observed 16 distinct variations in 78 viruses at neuraminidase antigenic sites, represented by NA residues 83–143, 156–190, 252–303, 330, 332, 340–345, 368, 370, 387–395, 400, 431–435, and 448–468 [17]. These variants were V83M (1); S95G (4); V106I (49); E119K (1); I163V (1); I188V (1); N189S (1); V264I (1); H275Y (5); S286G (16); E287K (1); N295S (1); T332A (2); S388L (1); V394I (17), and E462G (1) (summarized in Table S2). Most of these Canadian NA variant viruses were sampled during wave 1. Polymorphisms in Canada were numerous that delineated clades 5 through 7, namely NA V106I (49) variants and N248D (48); however, these also appeared early during the global pandemic [9]. Of note, all 16 S286G variants were clade 2 (36% of Canadian clade 2 viruses; *n* = 45 sampled) isolated during wave 1 in BC (1), MB (11), NFL (3), and PQ (1). Five of the Manitoba NA S286G variants were isolated during outbreak weeks 08, 09, and 11 and were associated with severe disease. The NA S286G variation was only seen with accompanying co-variation in PB1 (K480R), which is located in the region of PB1 that interacts with virus complementary RNA (cRNA) [18].

When analysis of positive selection was conducted, only two viral sites located in HA (namely D222G/E and E374K) displayed evidence of adaptive evolution, corresponding to the HA RBS and HA2-subunit position 47, respectively. Such sporadic drift in sampled Canadian viruses is encouraging from a public health perspective. It also agrees with prior studies demonstrating that little antigenic change occurs within a single influenza season and that genetic diversity within seasons is stochastic via chance introduction of divergent isolates [19]. Owing to HA antigenic and receptor binding site overlap, the biological significance of HA-222 mutations remains controversial [20]. However, HA-222 is a key determinant for virus binding to human-like receptors [14]. As mentioned, HA-374 is located proximal to a region identified in HA-neutralizing antibody structures that target a known antigenic site in the HA stem region [16]. Lastly, a number of co-varying amino acid substitutions were observed. As noted elsewhere, all NA S286G clade 2 virus variants from 4 regions (BC, MB, NFL, and PQ) co-varied with PB1 K480R. NA V394I co-varied with PB1 N158S in 15/16 clade 7 viruses from AB. In addition, HA Q293H (55 Canadawide) co-varied with HA K402T (37 Canadawide) in 36 clade 6 viruses in four regions (AB (1), MB (32), NB (2), and SK (1)).

One virus of note was RV0062-10/2009. A clade 7 virus, RV0062-10/2009 was collected during global outbreak week 36 in wave 2 (December 5, 2009) as a tracheal aspirate from a hospitalized case exhibiting severe respiratory illness. This virus genome revealed confirmed heterozygote bases that caused coding changes, namely, M1 Y240Y/F; M2 T11T/S; NA S35S/T, and PB2 D567D/G (Table S2). All variations are unique amongst the 235 sequenced A/H1N1pdm viruses sampled from Canada and to the best of our knowledge have not been reported elsewhere. The NA-35 mutation is located at the terminus of the transmembrane domain region associated with detergent-resistant lipid rafts [21]. Raft association may provide a critical determinant in targeting to apical membranes. NA-35 also is proximal to a presumably unstructured linker region (res 35–82) that connects the membrane anchor to the catalytic NA domain (residues 83 to 469) [17]. RV0062-10/2009 matrix protein mutations occur in the *C*-terminal fragment (165–254) of M1, which binds to vRNP [22], and the external domain of M2 *N*-terminal domain that also has been found to be under positive selection [23]. PB2 residues 538–676 form a compact, ordered domain with a novel fold [24]. PB2 residue 567 has previously been described as a major host specificity determinant site that distinguishes avian and swine from human isolates (Avian/Swine: charged residue aspartate (D); Human: uncharged residue asparagine (N) (1918 H1N1, 1957 H2N2, 1977 H3N2) or isoleucine (I) [25]. These heterozygous bases within RV0062-10/2009 imply that the associated infection, which was severe, was probably a mixed influenza A/H1N1pdm infection.

### A/H1N1pdm infection severity does not trend with virus genotype

As seen globally, A/H1N1pdm cases in Canada were predominantly experienced as mild respiratory illness (Table S1). Yet during Canada's wave 1, A/H1N1pdm laboratory-confirmed cases accounted for 1454 hospitalizations, 283 ICU admissions, and 72 fatalities [26] and during wave 2, A/H1N1pdm lab-confirmed cases accounted for 7081 hospitalizations, 1155 ICU admissions, and 347 deaths [27]. When normalized for population, the province of Manitoba (MB) and the Territory of Nunavut (NU) were particularly hard hit with A/H1N1pdm cases requiring hospitalizations (designated “severe”) in wave 1 [28] (Table S3; Source: FluWatch). Reasons for this are currently unknown [29]. Of the 158 (unique) viruses that were sequenced for which infection status was known, 26.5% (42/158) required hospitalization (Figure S1). Hence, to assess whether any particular virus signatures might account for influenza infection severity, we subjected the concatenated virus sequences to entropy-based sequence variability analysis. Owing to observed predominance in wave 2 of clade 7 viruses, we first independently analyzed wave 1 (*n* = 133) viruses (Table 2). Analysis specific to wave 2 could not be conducted owing to limited samples. A total of 6 A/H1N1pdm sequence locations (PB2-480; PB1-158; HA-203; HA-293; NA-394, and NS1-55) were observed with entropy differences greater than background; but only 4 were significantly correlated with clinical outcome as determined by Fisher Exact Test (in segment order: PB2-480; HA-203; NA-394, and NS1-55) (Table 2).

**Table 2:** Entropy difference, with randomization test, between severe and mild sequence populations for A/H1N1pdm viruses sampled in Canada (during Wave 1 only).

	Nucleotide Position^a^	Protein Position	AA Consensus (Mild/Severe)	AA Variance (Mild/Severe)	*P*-Value^b^	E-Score^c^
**01**	**PB2-1438**	**PB2-480**	**V (105/25)**	**I (1/3)**	**0.030**	**0.029**
02	PB1-473	PB1-158	N (92/28)	S (12/0)	0.010	0.069
**03**	**HA-604**	**HA-203**	**S (77/27)**	**T (28/1)**	**0.010**	**0.009**
04	HA-879	HA-293	Q (76/13)	H (28/11)	0.030	0.140
**05**	**NA-1180**	**NA-394**	**V (91/28)**	**I (14/0)**	**0.010**	**0.041**
**06**	**NS1-164**	**NS1-55**	**E (106/26)**	**G (0/2)**	**0.040**	**0.042**

a Nucleotide position on the coding sequence of each genome segment;

b *P* values were calculated from the randomization test on entropy score differences between severe and mild sequence populations;

c E-Scores were calculated from Fisher Exact Test; <0.05 highlighted in bold font.

Fewer HA-203 (T) variants were observed in the severe population (*P* = 0.009; Table 2). HA-S203T represents a clade 7-defining variation located near the HA monomer-monomer interface [12]. The small serine-to-threonine modification in side chain appears not to have had a dramatic effect on HA structure and introduction of the extra methylene group in the threonine side chain may assist in stabilizing the loop region in its surrounding environment [12]. Hence, perhaps the HA-203 mutation is advantageous for the virus and accounts for the observed early global predominance of clade 7 viruses.

No NA V394I variants were encountered in the severe population (*P* = 0.041; Table 2). NA V394I was present in 16 clade 7 viruses (AB) and one clade 3 virus (PQ). In contrast, no NS1-55 variants were detected in the mild case population (*P* = 0.042; Table 2); but only 3 total instances of this substitution were observed (RV1767/09 (case unknown; PQ; wk 6; clade 2); RV2018/09 (severe; MB; wk 9; clade 2); RV1975/09 (severe; MB; wk 9; clade 2)). Influenza A NS1 protein acts to suppress the host antiviral defenses at multiple levels and correlations between NS1 and virulence are documented [30]. Residue 55 is located within the third alpha-helix (residues 54–70) of the dsRNA-binding domain (RBD, residues 1–70) of NS1 [30]. Variation of NS1-55 from aspartate-to-glycine represents loss of a charged amino acid; thus, may stabilize the coiled-coiled helical structure.

Lastly, more PB2-480 (I) variants were detected in the severe population (*P* = 0.029; Table 2). PB2 residue 480 is located within the extreme carboxy-terminus of the cap-binding domain of PB2 (PB2_cap_: 318–483) [31]. PB2, along with PA, is involved in the generation of 5′-capped RNA primers through binding to and endonucleolytic cleavage of host pre-mRNAs. The same cap-binding domain also is required for PB2 binding interactions with various human chaperone complex proteins, such as: chaperonin CCT, stress-induced-phosphoprotein 1 (STIP1), Hsp70 and Hsp90, FK506 binding protein 5 (FKBP5), and alpha- and beta- tubulins [32]. Knockdown of CCT results in reduced growth of influenza A/WSN/33 virus demonstrating that CCT plays an important role in the virus life cycle, possibly by acting within a multimeric host chaperone complex to aid folding, assembly and transport of the virus RNA polymerase [32]. However, as only 5 total instances of this sporadic PB2-480 substitution were observed nationally, RV2735/09 (case unknown; AB; wk 19; clade 7) and the remainder regionally-isolated in MB [represented by RV1964/09 (mild; wk 8; clade 6); RV1977/09 (severe; wk 8; clade 6); RV1982/09 (severe; wk 8; clade 6) and RV2020/09 (severe; wk 9; clade 6)], the possibility of linked infections cannot be excluded.

Manitoba was particularly hard hit with community-acquired A/H1N1pdm infections [28], [29]. As such, the MB region was well sampled for virus sequencing and entropy-based sequence variability analysis. MB clade 6 viruses accounted for 40 viral sequences in our data set and 37.5% (15/40) required hospitalization, peaking during wave 1 in global outbreak weeks 9 and 10. MB clade 7 viruses accounted for 34 viral sequences in our data set, and 52.9% (18/34) required hospitalization, peaking in November during wave 2. Such intensified regional sampling may have introduced confounding bias in our severity analysis. Thus to avoid founder effects, entropy analysis also was conducted on the larger national data set for which infection status was known, representing 158 unique viruses (Figure S1). A total of 7 A/H1N1pdm sequence variant locations (PB2-526; PB1-587; PB1-158; HA-203; HA-383; NA-394, and NP-100) were observed with entropy differences greater than background; but only 4 were significantly correlated with clinical outcome as determined by Fisher Exact Test (in segment order: PB1-158; HA-203; NA-394, and NP-100) (Table S4). However, the predominance of clade 7 viruses that occurred in the wave 2 sampling period may have skewed this analysis. Aside from the co-varying clade 7-marker loci (HA-S203T and NP-V100I), we observed that PB1 N158S co-varied with NA V394I in a regional manner (15/16 of the Alberta NA V394I clade 7 viruses). Hence, we were unable to ascertain with confidence whether any particular A/H1N1pdm virus genotype trended with A/H1N1pdm infection severity in our nationwide survey.

## Discussion

On August 10 2010, the World Health Organization Director-General announced that the pandemic influenza A (A/H1N1pdm) novel reassortant virus had run its course and that the globe had entered into a post-pandemic period [33]. Unpredictability of viral mutations that arise during continued circulation in humans raises concerns regarding future severity of infections and potential for a continuing public health burden. Active molecular surveillance is important to monitor the pathogenicity of circulating strains, and to update pandemic preparedness and response plans accordingly. This study contributes whole-genome sequences for A/H1N1pdm viruses collected nationwide in Canada, providing additional perspective (relative to the larger global data set) of the molecular evolution of this emergent virus.

The first objective of our study was to acquire molecular insights into the trajectory of A/H1N1pdm following its introduction into Canada. Whole-genome sequences for 240 viruses (235 from unique subjects) collected across the country over 33 weeks were obtained, representing the largest national study undertaken to date. The chronological phylogeny for Canada revealed that five distinct A/H1N1pdm viral lineages entered and co-circulated in Canada in 2009. The Canadian A/H1N1pdm virus genomes revealed no evidence of further reassortment, and remained antigenically related to the clade 01 A/California 07/2009 isolate. Clade 2 viruses were waning in Canada by A/H1N1pdm global outbreak week 10; clade 6 viruses predominated in some regions during the peak period for the outbreak's wave 1; but thereafter, clade 7 viruses likely predominated nationwide (Figure 1; Table S2).

Early global predominance of clade 7 has been previously noted [9]. The clade 7 marker variation S203T (S220T in H1 propeptide), observed in 81 of the 235 sampled Canadian viruses, is located near the HA monomer-monomer interface [12]. Furuse *et al.* observed that position 206 (H3 numbering) is a A/H1N1pdm virus site under positive selection and also involved in antigenicity (Ca) [34]. In contrast, we did not find supporting evidence for positive selection at HA-203. Moreover, selection would be surprising in such a short time frame (∼8 months) given the limited immunity to A/H1N1pdm in the pre-vaccinated global population except for cross-reactive immunity observed in some elderly people [35].

The A/H1N1pdm virus has been simultaneously described as having a high genome-wide evolutionary rate (3.66×10^−3^ substitutions/site/year) [36] and a rate for the HA segment (0.9×10^−3^ substitutions/site/year) that represents the lowest of all of the viral HAs of seasonal H1N1 (1918–1957; 2.9×10^−3^), seasonal H1N1 (1977–2009; 1.7×10^−3^), and swine H1 (1930–2009; 1.9×10^−3^) [34]. However, elapsed time since A/H1N1pdm emergence is too limited (8 months in our sampling period to December 5, 2009) and precludes confident prediction of evolutionary rate trends for this virus. We observed a high degree of genetic conservation in terms of evolutionary distance (substitutions per site) within the Canadian whole-genome data set, which is in keeping with A/H1N1pdm virus emerging within an immune-naïve population. Genetic variation was highest in the segments encoding HA, NA and NP; however, most observed variation is explained by the fixed clade 2, 5, 6, and 7- defining marker variations (relative to ancestor sequences) in these three genes (up to 5 per virus), namely HA (K-15E [K2E], Q293H [Q310H]), and S203T [S220T]), NA (V106I and N248D), and NP (V100I); (residue positions within square-brackets for HA represent the H1 propeptide numbering scheme). In sum, our genome data set supports the conclusion that A/H1N1pdm is evolving but not excessively relative to other H1N1 influenza A viruses.

Among the Canadian sampled viruses, we observed elevated diversity in the PB1 segment, encoding RNA polymerase, that can only be partially explained by variants that circulated regionally [e.g., N158S (clade 7 - AB: 14 sequences); V157A (clade 2 - NS: 7 sequences); I181V and I535V (clade 2 - SK: 6 sequences); A587V (clade 2 - NS: 13 sequences)]. Highest variability was observed in clade 2 and clade 7 viruses within PB1 spanning residues 566 through 652; this PB1 region corresponds to the viral genomic RNA (vRNA)-binding domain (residues 493–757) [37]. PB1 diversity is intriguing given that A/H1N1pdm represents a reassortment of avian-like European swine genes (M1, M2, NA) with a triple-reassortment strain previously circulating in swine containing segments originally from the classical swine (NP, NS, HA), (H3N2) human-lineage PB1, and the avian-lineage PA and PB2 [36]. Previous studies have identified that compatibility among viral ribonucleoprotein (vRNP) complex proteins (NP, PA, PB1 and PB2) is necessary for optimal genome replication and transcription, and can be restrictive in reassortant influenza viruses [38]–[40]. Compensatory variations are often noted when a suboptimal vRNP complex is introduced into a new host species [18], [39], [41]. Although the A/H1N1pdm human-like PB1 gene and avian-like PB2 and PA genes have circulated in pigs since 1997–98 as triple reassortant swine virus [42], avian-like signatures remain for A/H1N1pdm PA and PB2 [25], suggestive of suboptimal replication in human hosts. Rapid pandemic spread of A/H1N1pdm may appear inconsistent with suboptimal host adaptation; however, antigenic novelty of A/H1N1pdm HA and NA proteins likely enabled the virus to overcome disadvantageous replication. Our observation hints that adaptive mutations in PB1 may compensate for suboptimal replication of the predominantly avian-like A/H1N1pdm polymerase complex.

It is worth noting that under new selective pressures, such as oseltamivir antiviral treatment, recombinant viruses with enhanced viral polymerase activity have been found capable of rapidly generating adaptive mutations that may enhance viral fitness [40]. Increased polymerase activity has been implicated in the extreme phenotype of the 1918 pandemic virus [43] and of avian viruses adapted to mammalian cells [44]. Enhanced viral polymerase activity also promotes replication and transcription of viral RNA leading to increased surface accumulation of HA and virus production [45]. Although we have no functional evidence to suggest increased polymerase activity (nor infection severity) associated with the detected Canadian PB1 variant viruses, further assessment of PB1 variations is warranted, both in the context of A/H1N1pdm transmission dynamics and virological properties.

Globally, 25% to 50% of severely ill A/H1N1pdm patients had no coexisting medical conditions [5], and in Canada many of the most ill were young, otherwise presumably healthy adults [29]. A second objective of this study was to interrogate whether any viral genome sites associated with increased infection severity. Although there has been speculation that certain sporadic A/H1N1pdm virus mutations may be associated with increased infection severity [46]–[48], we did not find evidence to support this hypothesis in our nationwide survey of circulating viruses over 33 weeks. For example, HA D222E/G/N (also called 239) and HA Q293H (also called 310) mutant viruses have been isolated worldwide from fatal laboratory-confirmed A/H1N1pdm cases [48]. As residue 222 (also called 239) is located within the HA receptor binding cavity, changes in this region could potentially influence virus binding and internalization into host cells [14]. However, we detected only 5 variants within the HA 219–240 region (all of unknown case status) during Canada's wave 2 (outbreak weeks 28, 29, 30, 32 and 36) in BC, NB, ON, and PQ. And of the total of 59 (24.9%) variants that were detected in the HA 301–316 region (wave 1 (51); wave 2 (8)), only 15 (25.4%) were associated with severe infections, 11 with unknown case severity, and the remainder (33) with mild cases. HA D222 mutations appear sporadically and spontaneously. And despite the fact that A/H1N1pdm HA D222E/G (and N) variant viruses have been detected in fatal cases, the same mutation also has been detected globally in mild A/H1N1pdm cases; conversely, viruses from numerous fatal cases lack the mutation. Thus, the clinical and public health significance of HA D222 variation remains unclear.

Notwithstanding the analysis of 133 unique wave 1 viruses and 158 wave 1 and 2 viruses (combined) for which infection status was known, our findings did not reveal any linkages between virus genotype and increased influenza infection severity. Although best attempts were made to achieve rigorous virus selection; lack of randomness in specimen sampling may have introduced bias (both temporal and geographic) that precluded us from achieving findings with significance. For example, we are aware that the sequenced Canadian viruses associated with severe A/H1N1pdm infections were not optimally distributed. Moreover, our entropy analysis was restricted given large numbers of specimens (*n* = 77) for which infection status remained unknown. Thus, benefits of a proactive and systematic process to link laboratory-generated molecular data and epidemiological data for enhanced surveillance purposes during such a pandemic were highlighted during the course of this study.

All sampled Canadian viruses encoded the A/H1N1pdm signature S31N (M2) allele that conveys antiviral resistance to Amantadine. The neuraminidase H275Y (H274Y in N2 numbering) mutation known to confer resistance to oseltamivir (Tamiflu®) and to peramivir, a drug used intravenously under an emergency use authorization, also was detected but only sporadically (*n* = 5) in 3 distinct clades/regions: namely clade 7 - AB (3); clade 3 - PQ (1) and clade 6 - ON (1). All resistant cases were associated with oseltamivir treatment. Such low frequency resistance is reassuring given the pervasive application in Canada of antivirals in clinically-diagnosed A/H1N1pdm cases. This H275Y mutation does not cause resistance to zanamivir (Relenza®). Although recent data suggests that oseltamivir-resistance does not alter viral fitness and may induce severe disease at least in ferrets [49], the 5 cases we detected (mild (2) and unknown (3)) contribute no further insight on this issue.

Complete genome sequencing of influenza A viruses is essential to determine the genetic basis of pathogenicity, antiviral resistance, and to better understand viral evolution. We reconstructed whole-genome sequences by conventional Sanger-based sequencing of overlapping amplicons yielding a consensus assembly of the sequence reads for each viral segment. However, influenza A virus mutation rates during replication are estimated at ∼1 nucleotide change per 10,000 nucleotides [50] and most influenza infections have 10–1000 associated virions possessing varying nucleotides in their genomes, resulting in the generation of a viral “quasispecies” in which closely related but non-identical recombinant viral genomes may co-circulate within a single host [3]. We acknowledge that A/H1N1pdm consensus sequencing will describe only the most dominant viral variant(s) within an intra-host population. Therefore, other important intra-host genetic diversity (quasispecies and mixed infections) may be underrepresented with this conventional approach, and may account for why only one example of a potential mixed infection during wave 2 (RV0062-10/2009) was detectable. In some cases, wild type sequence has been detected in nasopharyngeal swabs at the same time that hemagglutinin sequence variants, particularly at HA residue 222 (also known as 225: H3 numbering), have been reported in deeper lung specimens from the same patient [46], [51]. Mixed infections with multiple virus types can lead to reassortment and may have a significant role in virus evolution. Furthermore, as more diverse influenza viruses circulate with time and mixed infections increase, potential for reassortment increases; thus, the adaptive evolutionary rate of A/H1N1pdm can be expected to accelerate with potentially important public health consequences [52]. It remains important to follow viral evolutionary trends, preferably applying newer higher density sequencing technologies in order to detect emerging variants with enhanced sensitivity and timeliness.

Our results (and those of others) underscore the complexity of influenza A virus genetics and advocate for intensified global surveillance applying whole-genome sequence data. Although evaluating effects of vaccination on viral evolutionary trends and vaccine efficacy over time are important, this study was not designed to address such issues. Continued molecular surveillance studies are indeed warranted to monitor A/H1N1pdm virus genotypic and phenotypic diversity in the post-vaccination era, providing information to enable timely epidemic or pandemic detection, inform health policy decisions and influenza management strategies, and to guide influenza vaccine development and prioritization. With the advent of worldwide sentinel surveillance in humans and animals as well as advances made in rapid whole-genome sequencing, mathematical modeling, global alerting, immunopathogenicity, and drug and vaccine therapeutics, public health officials now have assembled necessary tools to intervene effectively to prevent major morbidity and mortality from influenza disease. Fortunately, this marks a major turning point in the approach to management of influenza.

## Materials and Methods

### Viral culture

Virus specimens were collected from subjects with influenza-like illnesses as part of the ongoing Canadian influenza surveillance program. This program continuously monitors genetic and antigenic changes in influenza viruses circulating in Canada. As a national public health surveillance program, informed consent from subjects is not required. However, as some additional specimens were collected nationally from subjects that required hospitalization and participated in specific H1N1-related studies, ethics approval was acquired from the University of Manitoba Health Ethics Board to investigate these viruses. For these patients, written informed consent was obtained from each patient or their surrogate. Specimens originated from eleven of the twelve provinces and territories of Canada, namely Alberta (AB), British Columbia (BC), Manitoba (MB), New Brunswick (NB), Newfoundland and Labrador (NFL), Nova Scotia (NS), Northwest Territories (NWT), Nunavut (NU), Ontario (ON), Quebec (PQ), and Saskatchewan (SK); missing: Yukon. For virus propagation, 50 µL of clinical specimens was inoculated into the Madin-Darby canine kidney cell line (MDCK) (CCL34; American Type Culture Collection, Rockville, Md.) and examined for cytopathic effect. The MDCK cells were maintained in Eagles's MEM containing 100 U/mL Penicillin, 100 µg/mL Streptomycin, 0.292 mg/mL Glutamine, 25 mM Hepes buffer, 0.1 mM MEM non essential amino acid, 1 mM MEM sodium pyruvate and 2 µg/mL TCPK-trypsin. Cells were incubated at 37°C in a CO_2_ incubator and observed daily for cytopathic effect. Influenza A/H1N1pdm virus was confirmed by molecular evaluation (i.e., real-time RT-PCR) and antiviral sensitivities inferred from DNA sequences were confirmed by phenotypic analyses.

### Virus sequencing

A sum total of 240 A/H1N1pdm culture- and amplification- positive clinical specimens were collected from 235 distinct individuals and subjected to Sanger sequencing to generate consensus assemblies for each virus segment (Table S1). A total of 178 unique, consensus whole-genome sequences were derived for A/H1N1pdm specimens from Canada's wave 1 (defined as April 16 to August 29 2009, inclusive); whereas 57 unique virus genomes were acquired for Canada's wave 2 (August 30 2009 to December 5 2009 herein). These specimens were associated with mild to severe (requiring hospitalization) influenza infections, when known (*n* = 158), and were distributed geographically and temporally (Table S1).

The first A/H1N1pdm virus infection encountered in Canada was sampled on April 16 2009 in Quebec (PQ), representing week 03 of the global outbreak (Figure S1; Table S1). Although regional variation in laboratory-confirmed A/H1N1pdm cases occurred, the nationwide incidence peak for wave 1 occurred during global outbreak weeks 12 to 14 for most regions (range, 8-18) (Figure S2; Table S5) [26]. The nationwide incidence peak for wave 2 occurred predominantly during global outbreak week 32 (range, 31–34) (Figure S2; Table S5) [27]. Thereafter, influenza activity remained low through the beginning of 2010 (Figure S2); and on January 27 2010 PHAC announced that wave 2 of pandemic A/H1N1 2009 in Canada had tapered off [27].

The influenza A (H1N1) 2009 vaccine produced for Canada by GlaxoSmithKline, called Arepanrix H1N1, was approved on October 21 2009; and vaccinations began for high-risk groups on October 26 2009 (representing week 31 of the global outbreak), with the general public following thereafter (Figures S1 and S2). Although vaccination status was unknown at sampling, only 17 of the 240 sequenced viruses were sampled after initiation of Canada's vaccination campaign (including 2 redundantly-sampled, severe A/H1N1pdm patients; Figure S1). However, only 11.6 million or 41% of Canadians (excluding the territories) aged >12 years received vaccination during the entire A/H1N1pdm influenza campaign, with varying rates across the country according to new self-reported data from the 2010 Canadian Community Health Survey [53].

Where noted, specimens received as primary specimens or Tissue Culture Fluid (TCF) extracts were directly processed. Otherwise, viruses were first propagated in MDCK cells, as described. Viral RNA was extracted, in the presence of supplied carrier RNA, with the MagMAX™ Viral RNA Isolation Kit (Applied Biosystems, Foster City CA) using a MagMAX™ Express-96 Magnetic Particle Processor according to the manufacturer's instructions. Extractions were performed on 140 µL for the cultures and 265 µL for the primary specimens. Complementary DNA was synthesized by reverse transcription reaction, and amplification by PCR was performed using specific primers for each gene segment for a sum of 14 amplicons. RT-PCR reactions were generated using the OneStep RT-PCR kit (Qiagen Inc., Mississauga, Canada) with either the universal influenza A generic primer, Uni12, as reported elsewhere [54] or with A/H1N1pdm-specific primers (Table S6). Reactions were performed using BigDye-Terminator™ v3.1 Cycle Sequencing Reaction Kit on a 3730 xl Genetic Analyzer (Applied Biosystems, Foster City, CA, USA) following the manufacturer's instructions. The A/H1N1pdm viruses were bi-directionally sequenced across all segments using 52 primer sets; the f1 and r1 primers for each segment target the 5′-start and 3′-end of the coding region, respectively (Table S6). Remaining primers in the series are distributed at approximately 200 to 500-bp intervals. Sequences were assembled, curated, and edited using Lasergene® v8.1 SeqMan Pro software (DNASTAR Inc., Madison, USA). RNA was extracted from a total of 240 viruses sampled in Canada from April 16 2009 to December 5 2009 (33 weeks) (Table S1). Passage histories of the sequenced viruses are noted; A/H1N1pdm virus propagation by cell culturing was required in all except 42 cases. Five samples were redundantly sampled from the same individuals to compare virus sequences: from primary specimens (designation, p) versus cultured virus isolates (designation, i) (RV2076/09; RV2042/09, and RV2066/09) or from two distinct anatomical sites [RV2006-10/2009 (nasopharyngeal; NP) versus RV2007-10/2009 (tracheal; TRA); RV2010-10/2009 (NP) versus RV2011-10/2009 (TRA)]; no nucleotide differences were detected, indicating that the A/H1N1pdm virus sequences were conserved. Hence, a sum total of 235 unique whole-genome sequences were derived from unique human subjects. Full genome sequences of all viruses sequenced (*n* = 240) are available for download (accession numbers listed in Table S7).

### Phylogenetic analysis

In addition to the Canadian human-isolated A/H1N1pdm virus genomes sequenced in this study, other full-length sequences (10 coding sequences) available as of April 1 2009 to June 2 2010 (inclusive) (*n* = 1198) sequences were downloaded from the influenza sequence database (Influenza Virus Resource: http://www.ncbi.nlm.nih.gov/genomes/FLU/FLU.html, accessed June 2, 2010). Accession numbers for the downloaded global genome references are provided in Tables S8 and S9. Ten coding regions for the eight genome segments for each virus were concatenated by an in-house developed Perl script. Nucleotide alignments were constructed using ClustalW [55] followed by manual alignment to codon position in Molecular Evolutionary Genetics Analysis (MEGA) ver. 4.1 [56]. Phylogenetic trees were inferred using the neighbour-joining distance method, with genetic distances calculated using Kimura's two-parameter model (K2P and bootstrapping test) in MEGA 4.0. Confidence values for the branches were provided by bootstrap analysis of 1000 replicates. Genetic Algorithm for Rapid Likelihood Inference (GARLI) ver. 0.96 (http://www.nescent.org/informatics/download.php?software_id=4, accessed June 2, 2010) also was applied to infer or assess the reliability of the phylogenetic trees [57]. In this method, rate variation across sites was estimated from a gamma-distribution with non-parametric bootstrapping at 100 replicates. The resultant consensus tree was generated using the Summarization of Split Support on Phylogenetic Trees (SumTrees) program ver. 1.02, which is part of the DendroPy Phylogenetic Computation Library ver. 2.6.1 [58]. Classification of representative clades were based primarily on the classifications for related sequences reported previously [9] [59] but also on the bootstrapping values (>70%) for distinguished branch lengths. Phylogenetic trees were displayed using MEGA 4.0 and TreeView 1.6.6 [60] and inspected manually.

### Sequence analysis

Mutations within A/H1N1pdm genes were determined by Lasergene® v8.1 SeqMan Pro software (DNASTAR Inc., Madison, USA) and displayed by Jalview v2.4.0 [61]. Identified coding mutations within A/H1N1pdm genes of the Canadian viruses were mapped to previously reported hemagglutinin antigenic sites (Sa, Sb, Ca1/2, and Cb) [11] and neuraminidase antigenic sites [17]. Genetic diversity of A/H1N1pdm genes was estimated by Maximum Composite Likelihood (nucleotide) and Dayhoff (amino acid) models in MEGA 4.0 [56]. An entropy approach was used to compare two sequence populations and identify the significant (*P*<0.05) amino acid differences with randomization test [62]. A Fisher Exact Test was applied to infer whether associations between genetic variations and A/H1N1pdm disease were statistically supported. Sites exhibiting co-evolutionary associations were predicted by a Bayesian model, using SPIDERMONKEY [63].

### Identification of positively selected sites

A/H1N1pdm sites exhibiting positive selection were identified using the codon-based maximum likelihood method, Fixed Effects Likelihood (FEL) within the Hypothesis Testing Using Phylogenies (HyPhy) package [64]. This was used to test the hypothesis as to whether relative rates of nonsynonymous and synonymous substitutions (dN/dS) at a given site differed between two datasets along the phylogenetic tree. Before this analysis, an evolutionary model was first estimated to fit the analyzed aligned sequences by the Codon Model Selection (CMS) module.

#### Accession Numbers

Viral sequences from this study have been deposited into GenBank (accession numbers in Supplementary Information, Table S7). Accession numbers for sequences from global sources that were used in the generation of Figure 2 and 4 trees are listed in Table S8; accession numbers for sequences from global sources that were used in the clade assignment for Figure 3 are listed in Table S9.

#### Perl scripts

The in-house developed Perl scripts are available upon request.

## Supporting Information

Figure S1 **Regional and temporal distribution of A/H1N1pdm viruses sequenced from Canadian provinces and territories.** X-axis displays the temporal distribution of sampled viruses according to global outbreak week. Global outbreak week 01 includes April 1 2009, the date of collection for the first global A/H1N1pdm virus. Y-axis displays the Canadian regional distribution; abbreviations: Alberta (AB), British Columbia (BC), Manitoba (MB), New Brunswick (NB), Newfoundland and Labrador (NFL), Nova Scotia (NS), Nunavut (NU), Northwest Territories (NWT), Ontario (ON), Quebec (PQ), and Saskatchewan (SK). Distribution of infection case status: When known, the infection status associated with each virus is depicted with coloured spheres: teal (mild); severe (red). Otherwise, unknown case status is depicted with triangles (black).
(TIF)

Figure S2 **Regional and temporal distribution of A/H1N1pdm infections in Canada.** A. National case incidence according to the global outbreak initiated April 1 2009, corresponding to the date of collection for the first global A/H1N1pdm virus. B. Regional incidence for Canada during Wave 1. C. Regional incidence for Canada during Wave 2.
(TIF)

Table S1 Descriptions for A/H1N1pdm viruses sampled in Canada.
(XLS)

Table S2 Amino acid variations and clades detected for A/H1N1pdm viruses sampled in Canada.
(XLS)

Table S3 Cumulative hospitalized A/H1N1pdm cases in Canada during Wave 1, by region.
(DOC)

Table S4 Entropy difference, with randomization test, between severe and mild sequence populations for A/H1N1pdm viruses sampled in Canada (Wave 1 and 2, inclusive).
(DOC)

Table S5 Summary of A/H1N1pdm infection incidence in Canada.
(DOC)

Table S6 Oligonucleotides used to amplify and sequence A/H1N1pdm viruses in this study.
(DOC)

Table S7 Accession numbers for Influenza A/H1N1pdm viruses sequenced in this study.
(DOC)

Table S8 Accession numbers for the global outbreak A/H1N1pdm virus reference sequences used for generating Figures 2 and 4.
(DOC)

Table S9 Accession numbers for the global outbreak A/H1N1pdm virus reference sequences used for clade assignment in Figure 3.
(DOC)
