# Supplementary figures and images for: Nationwide Molecular Surveillance of Pandemic H1N1 Influenza A Virus Genomes: Canada, 2009
Source: PLoS One. 2011 Jan 7;6(1):e16087. doi: 10.1371/journal.pone.0016087 (PMC3017559; doi:10.1371/journal.pone.0016087)

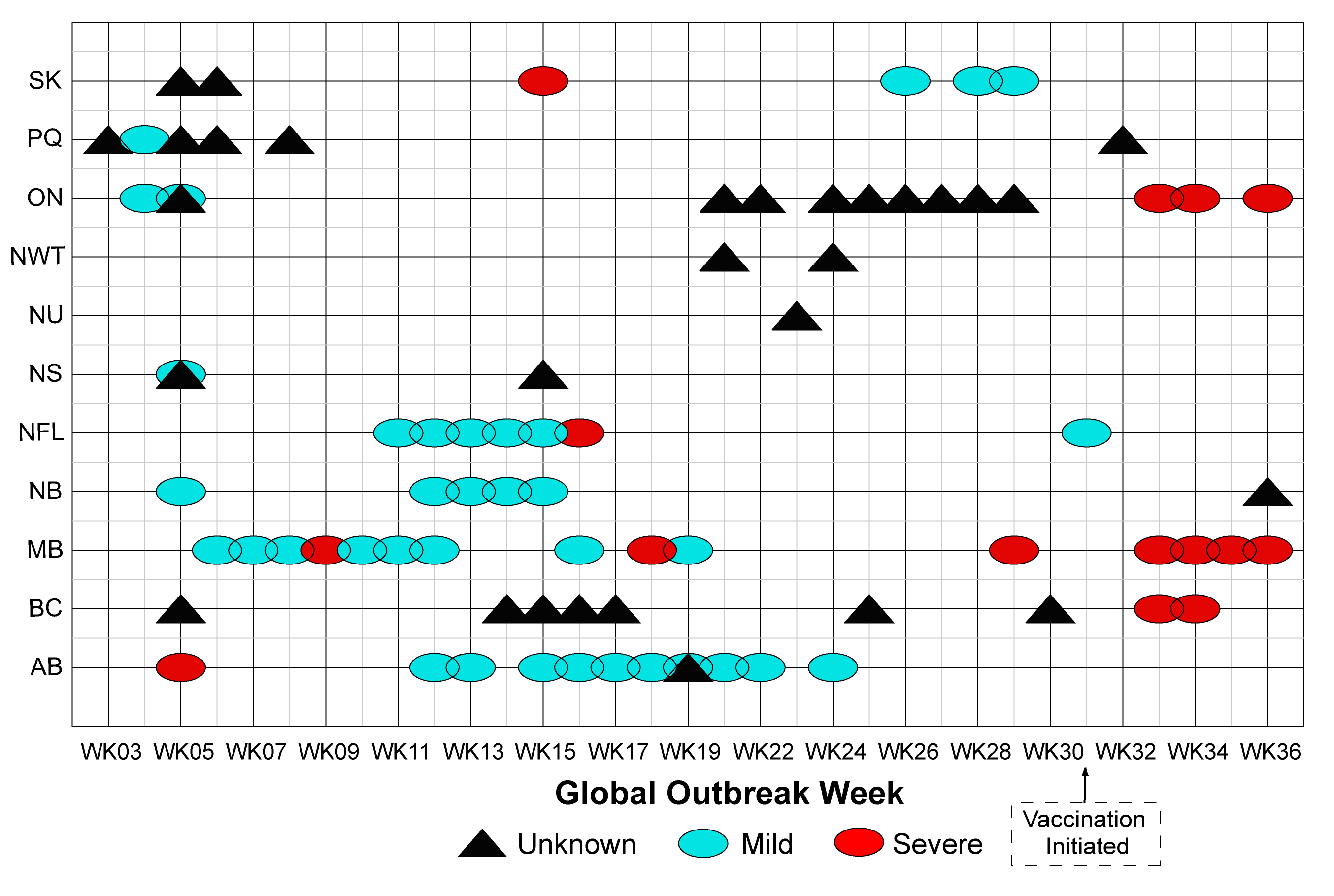

Supplement: Figure S1 — Regional and temporal distribution of A/H1N1pdm viruses sequenced from Canadian provinces and territories. X-axis displays the temporal distribution of sampled viruses according to global outbreak week. Global outbreak week 01 includes April 1 2009, the date of collection for the first global A/H1N1pdm virus. Y-axis displays the Canadian regional distribution; abbreviations: Alberta (AB), British Columbia (BC), Manitoba (MB), New Brunswick (NB), Newfoundland and Labrador (NFL), Nova Scotia (NS), Nunavut (NU), Northwest Territories (NWT), Ontario (ON), Quebec (PQ), and Saskatchewan (SK). Distribution of infection case status: When known, the infection status associated with each virus is depicted with coloured spheres: teal (mild); severe (red). Otherwise, unknown case status is depicted with triangles (black). (TIF) [file pone.0016087.s001.tif]

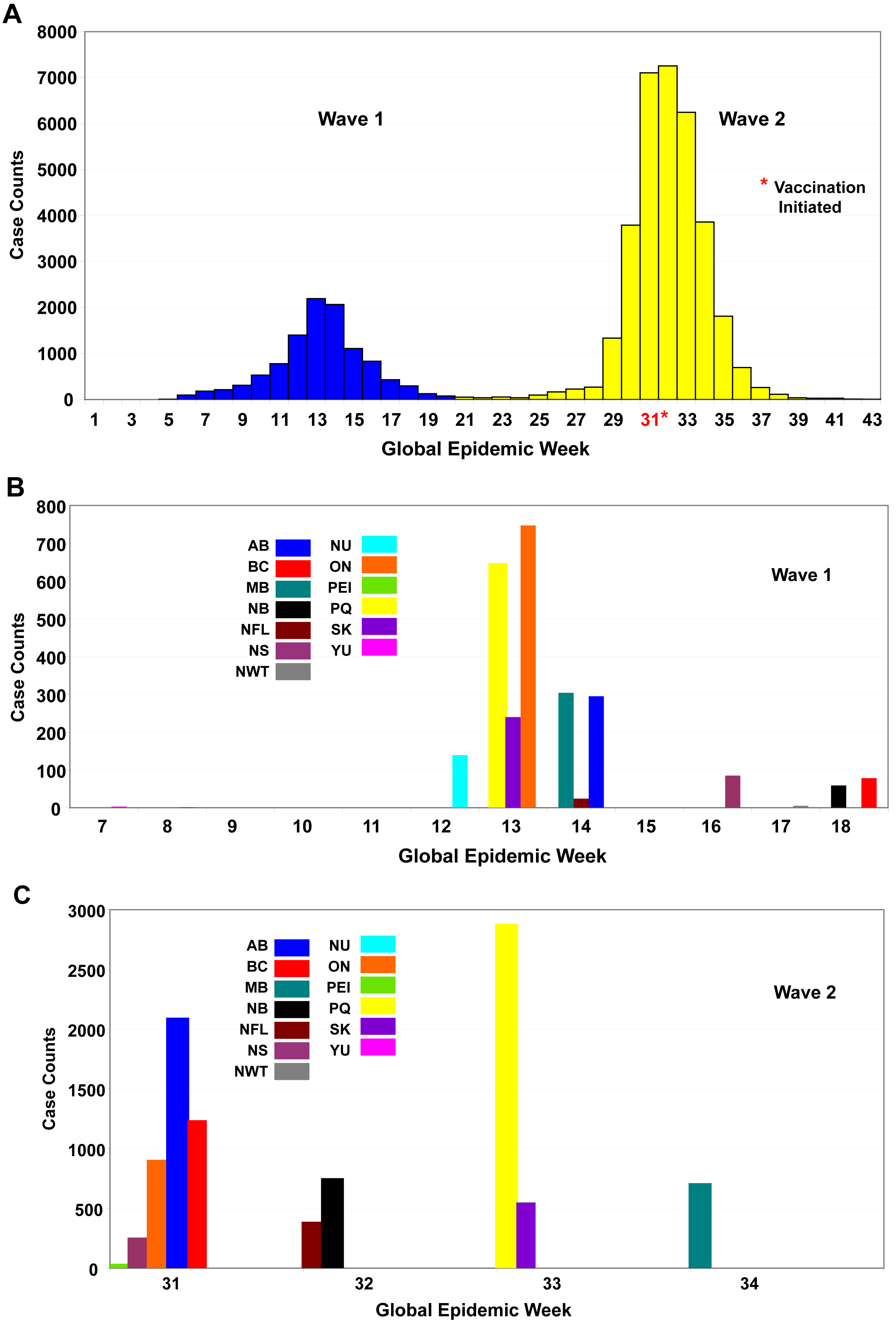

Supplement: Figure S2 — Regional and temporal distribution of A/H1N1pdm infections in Canada. A. National case incidence according to the global outbreak initiated April 1 2009, corresponding to the date of collection for the first global A/H1N1pdm virus. B. Regional incidence for Canada during Wave 1. C. Regional incidence for Canada during Wave 2. (TIF) [file pone.0016087.s002.tif]
