# Supplementary material for: Nationwide Molecular Surveillance of Pandemic H1N1 Influenza A Virus Genomes: Canada, 2009
Source: PLoS One. 2011 Jan 7;6(1):e16087. doi: 10.1371/journal.pone.0016087 (PMC3017559; doi:10.1371/journal.pone.0016087)
Supplement: Table S3 — Cumulative hospitalized A/H1N1pdm cases in Canada during Wave 1, by region. (DOC) [file pone.0016087.s005.doc]

**Table S3.** Cumulative hospitalized A/H1N1pdm cases in Canada during Wave 1, by region

|  | **Cumulative Hospitalized A/H1N1pdm Cases** **^a^** |
| --- | --- |
| AB | 127 |
| BC | 42 |
| **MB** | **221** |
| NB | 2 |
| NFL | 3 |
| NS | 17 |
| **NU** | **62** |
| NWT | 4 |
| ON | 361 |
| PEI | 1 |
| PQ | 591 |
| SK | 23 |
| YT | 0 |

^a^ As reported by Canada’s national sentinel surveillance system (FluWatch) weeks 16 to 34 (inclusive; FluWatch 2008/2009 season), corresponding to Global Outbreak Weeks 04 to 22. (Source: <http://www.phac-aspc.gc.ca/fluwatch/08-09/w34_09/index-eng.php>).

^b^ Regions with elevated A/H1N1pdm-associated hospitalizations (severe infections) following normalization for regional population are denoted in boldface.
