# Supplementary material for: Nationwide Molecular Surveillance of Pandemic H1N1 Influenza A Virus Genomes: Canada, 2009
Source: PLoS One. 2011 Jan 7;6(1):e16087. doi: 10.1371/journal.pone.0016087 (PMC3017559; doi:10.1371/journal.pone.0016087)
Supplement: Table S4 — Entropy difference, with randomization test, between severe and mild sequence populations for A/H1N1pdm viruses sampled in Canada (Wave 1 and 2, inclusive). (DOC) [file pone.0016087.s006.doc]

**Table S4.** Entropy difference, with randomization test, between severe and mild sequence populations for A/H1N1pdm viruses sampled in Canada (Wave 1 and 2, inclusive).

|  | **Nucleotide Position^a^** | **Protein position** | **AA Consensus (Mild/Severe)** | **AA Variance**  **(Mild/Severe)** | ***P*-Value^b^** | **E-Score^c^** |
| --- | --- | --- | --- | --- | --- | --- |
| 01 | PB2-1577 | PB2-526 | K (111/44) | R (6/0) | 0.03 | 0.287 |
| **02** | **PB1-473** | **PB1-158** | **N (103/44)** | **S (12/0)** | **0** | **0.038** |
| 03 | PB1-1760 | PB1-587 | V (108/44) | A (6/0) | 0 | 0.277 |
| **04** | **HA-604** | **HA-203** | **S (78/42)** | **T (38/2)** | **0** | **<0.0001** |
| 05 | HA-1144 | HA-382 | I (111/44) | V (6/0) | 0.03 | 0.19 |
| **06** | **NA-1180** | **NA-394** | **V (102/44)** | **I (14/0)** | **0** | **0.012** |
| **07** | **NP-298** | **NP-100** | **I (82/38)** | **V (33/6)** | **0.01** | **0.042** |

^a^ nucleotide position on the coding sequence of each genome segment

^b^ *P* values were calculated from the randomization test on entropy score differences between severe and mild sequence populations

^c^ E-Scores were calculated from Fisher Exact Test; < 0.05 are denoted in boldface

^d^ Wave 1, defined as epidemic week starting April 13, 2009 to August 24, 2009, inclusive. Wave 2, defined as epidemic week starting August 31, 2009 to January 22, 2010, inclusive.
