# Supplementary material for: Nationwide Molecular Surveillance of Pandemic H1N1 Influenza A Virus Genomes: Canada, 2009
Source: PLoS One. 2011 Jan 7;6(1):e16087. doi: 10.1371/journal.pone.0016087 (PMC3017559; doi:10.1371/journal.pone.0016087)
Supplement: Table S5 — Summary of A/H1N1pdm infection incidence in Canada. (DOC) [file pone.0016087.s007.doc]

**Table S5.**  Summary of A/H1N1pdm infection incidence in Canada.

| **GO Week^a,b^** | **Week Starting** | **AB** | **BC** | **MB** | **NB** | **NFL** | **NS** | **NU** | **NWT** | **ON** | **PEI** | **PQ** | **SK** | **YT** |
| --- | --- | --- | --- | --- | --- | --- | --- | --- | --- | --- | --- | --- | --- | --- |
| 01 | 30-Mar | 0 | 0 | 0 | 0 | 0 | 0 | 0 | 0 | 0 | 0 | 0 | 0 | 0 |
| 02 | 6-Apr | 0 | 0 | 0 | 0 | 0 | 0 | 0 | 0 | 0 | 0 | 0 | 0 | 0 |
| 03 | 13-Apr | 0 | 0 | 0 | 0 | 0 | 0 | 0 | 0 | 0 | 0 | 1 | 0 | 0 |
| 04 | 20-Apr | 0 | 0 | 0 | 0 | 0 | 0 | 0 | 0 | 0 | 0 | 0 | 0 | 0 |
| 05 | 27-Apr | 0 | 2 | 0 | 0 | 0 | 4 | 0 | 0 | 0 | 0 | 0 | 0 | 0 |
| 06 | 04-May | 18 | 27 | 1 | 1 | 0 | 29 | 0 | 0 | 16 | 0 | 3 | 0 | 0 |
| 07 | 11-May | 30 | 50 | 0 | 1 | 0 | 23 | 0 | 0 | 60 | 3 | 12 | 4 | 0 |
| 08 | 18-May | 19 | 21 | 3 | 0 | 0 | 10 | 0 | 0 | 111 | 0 | 32 | 15 | **1** |
| 09 | 25-May | 27 | 15 | 2 | 0 | 0 | 4 | 0 | 0 | 107 | 0 | 96 | 58 | 0 |
| 10 | 01-Jun | 27 | 7 | 5 | 0 | 0 | 3 | 1 | 0 | 332 | 0 | 110 | 46 | 0 |
| 11 | 08-Jun | 29 | 20 | 29 | 0 | 0 | 2 | 5 | 2 | 452 | 0 | 184 | 56 | 0 |
| 12 | 15-Jun | 90 | 25 | 79 | 0 | 9 | 4 | **139** | 0 | 703 | 0 | 269 | 91 | 0 |
| 13 | 22-Jun | 258 | 42 | 176 | 1 | 7 | 19 | 59 | 3 | **747** | 0 | **643** | **240** | 0 |
| 14 | 29-Jun | **295** | 55 | **304** | 6 | **24** | 32 | 54 | 2 | 633 | 0 | 485 | 175 | 0 |
| 15 | 06-Jul | 278 | 34 | 86 | 1 | 4 | 41 | 82 | 2 | 303 | 2 | 186 | 89 | 0 |
| 16 | 13-Jul | 156 | 5 | 102 | 13 | 7 | **85** | 48 | 0 | 172 | 0 | 172 | 74 | 0 |
| 17 | 20-Jul | 121 | 70 | 44 | 19 | 2 | 74 | 17 | **5** | 0 | 0 | 67 | 11 | 0 |
| 18 | 27-Jul | 30 | **78** | 9 | **59** | 16 | 45 | -- | -- | 41 | **3** | 0 | 15 | -- |
| 19 | 03-Aug | 16 | 36 | 10 | 7 | 17 | 26 | -- | -- | 13 | 1 | 0 | 2 | -- |
| 20 | 10-Aug | 12 | 30 | 1 | 3 | 4 | 8 | -- | -- | 9 | 2 | 0 | 6 | -- |
| 21 | 17-Aug | 4 | 14 | 1 | 4 | 4 | 3 | -- | -- | 12 | 0 | 0 | 8 | -- |
| 22 | 24-Aug | 4 | 20 | 3 | 0 | 4 | 0 | -- | -- | 4 | 1 | 0 | 2 | -- |
| 23 | 31-Aug | 34 | 9 | 1 | 0 | 1 | 0 | -- | -- | 3 | 1 | 0 | 7 | -- |
| 24 | 07-Sep | 18 | 13 | 0 | 1 | 0 | 2 | -- | -- | 1 | 0 | 0 | 3 | -- |
| 25 | 14-Sep | 39 | 50 | 0 | 1 | 0 | 2 | -- | -- | 4 | 0 | 0 | 2 | -- |
| 26 | 21-Sep | 56 | 91 | 0 | 0 | 1 | 1 | -- | -- | 9 | 1 | 0 | 5 | -- |
| 27 | 28-Sep | 69 | 129 | 0 | 0 | 0 | 2 | -- | -- | 24 | 0 | 0 | 2 | -- |
| 28 | 05-Oct | 146 | 12 | 3 | 0 | 0 | 4 | -- | -- | 66 | 0 | 33 | 5 | -- |
| 29 | 12-Oct | 403 | 617 | 11 | -- | 6 | 3 | -- | -- | 162 | 3 | 84 | 46 | -- |
| 30 | 19-Oct | 1383 | 1170 | 19 | 7 | 69 | 63 | -- | -- | 490 | 7 | 492 | 92 | -- |
| 31 | 26-Oct | **2082** | **1239** | 37 | 67 | 283 | **241** | -- | -- | **892** | **37** | 1785 | 443 | -- |
| 32 | 02-Nov | 669 | 1153 | 218 | **739** | **374** | 143 | -- | -- | 760 | 26 | 2668 | 505 | -- |
| 33 | 09-Nov | 444 | 717 | 258 | 594 | 127 | 138 | -- | -- | 544 | 8 | **2867** | **550** | -- |
| 34 | 16-Nov | 211 | 345 | **696** | 257 | 64 | 116 | -- | -- | 317 | 9 | 1475 | 369 | -- |
| 35 | 23-Nov | 81 | 153 | 360 | 121 | 17 | 20 | -- | -- | 117 | 1 | 773 | 170 | -- |
| 36 | 30-Nov | 62 | 49 | 97 | 29 | 6 | 14 | -- | -- | 66 | 3 | 307 | 63 | -- |
| 37 | 07-Dec | 30 | 25 | 30 | 11 | 3 | 1 | -- | -- | 28 | 0 | 107 | 28 | -- |
| 38 | 14-Dec | 15 | 13 | 28 | 2 | 0 | 0 | -- | -- | 18 | 0 | 34 | 3 | -- |
| 39 | 21-Dec | 6 | 6 | 9 | 3 | 0 | 1 | -- | -- | 6 | 0 | 5 | 3 | -- |
| 40 | 28-Dec | 2 | 5 | 14 | 1 | 0 | 0 | -- | -- | 1 | 0 | 4 | 1 | -- |
| 41 | 04-Jan | 3 | 6 | 6 | 0 | 0 | 0 | -- | -- | 8 | 0 | 5 | 1 | -- |
| 42 | 11-Jan | 2 | 1 | 0 | 1 | 0 | 0 | -- | -- | 4 | 0 | 4 | 0 | -- |
| 43 | 18-Jan | 0 | 0 | 0 | 1 | 0 | 0 | -- | -- | 6 | 0 | 0 | 0 | -- |

^a^ GO Week, denotes global outbreak week. Corresponds to FluWatch calendar weeks for Canada’s national sentinel surveillance system (FluWatch, 2008-2009 season).

^b^ Wave 1, defined as epidemic week starting April 13, 2009 to August 24, 2009, inclusive. Regional incidence peaks are denoted in boldface.

^c^ Wave 2, defined as epidemic week starting August 31, 2009 to January 22, 2010, inclusive. Regional incidence peaks are denoted in boldface.

^d^ --, denotes missing data.
