# Supplementary material for: Nationwide Molecular Surveillance of Pandemic H1N1 Influenza A Virus Genomes: Canada, 2009
Source: PLoS One. 2011 Jan 7;6(1):e16087. doi: 10.1371/journal.pone.0016087 (PMC3017559; doi:10.1371/journal.pone.0016087)
Supplement: Table S6 — Oligonucleotides used to amplify and sequence A/H1N1pdm viruses in this study. (DOC) [file pone.0016087.s008.doc]

**Table S6**. Oligonucleotides used to amplify and sequence A/H1N1pdm viruses in this study

| **Primer** | **Sequence** | **Coordinates ^a^** | **Orientation** |
| --- | --- | --- | --- |
| swHAf | CTAGTGGTACCGAGATATGC | 793-812 | + |
| swHAf1 | ATGAAGGCAATACTAGTAGT | 1-20 | + |
| swHAf2 | AATTCATACCCAAAGCTCAG | 517-536 | + |
| swHAf3 | CACAGAATGCCATTGACGAG | 1154-1173 | + |
| swHAr | TTCTGAAATGGGAGGCTGGT | 913-932 | - |
| swHAr1 | TTAAATACATATTCTACACT | 1682-1701 | - |
| swHAr2 | AACTCTTTACCTACTGCTGT | 1222-1241 | - |
| swHAr3 | CACTAGTAGATGGATGGTGA | 588-607 | - |
| swMPf1 | ATGAGTCTTCTAACCGAGGT | 1-20 | + |
| swMPf2 | TTGGTCTAGTGTGTGCCACT | 431-450 | + |
| swMPr1 | TTACTCTAGCTCTATGTTGA | 963-982 | - |
| swMPr2 | GTAGTAGCCATCTGTCTGTG | 484-503 | - |
| swNAf1 | ATGAATCCAAACCAAAAGAT | 1-20 | + |
| swNAf2 | GATTTGAGTCAGTCGCTTGG | 518-537 | + |
| swNAf3 | AAGACAGGCAGTTGTGGTCC | 991-1010 | + |
| swNAr1 | TTACTTGTCAATGGTAAATG | 1391-1410 | - |
| swNAr2 | TATCCAAACACCATTGCCGT | 1058-1077 | - |
| swNAr3 | AGAAATTCCAATTGTTAGCC | 569-588 | - |
| swNPf1 | ATGGCGTCTCAAGGCACCAA | 1-20 | + |
| swNPf2 | ATCCCAGAATGTGCTCTCTA | 479-498 | + |
| swNPf3 | TGGCATGCCACTCTGCTGCA | 992-1011 | + |
| swNPr1 | TCAACTGTCATACTCCTCTG | 1478-1497 | - |
| swNPr2 | TCTTGTGGAAAGCTTTCCTC | 1064-1083 | - |
| swNPr3 | TCACCGCAGCACCTGCGGCA | 531-550 | - |
| swNSf1 | ATGGACTCCAACACCATGTC | 1-20 | + |
| swNSf2 | CACTGAGGAGGGAGCAATAG | 450-469 | + |
| swNSr1 | GTAGAAACAAGGGTGTTTTT | 844-863 | - |
| swNSr2 | GACATCCTCATAAGTATGTC | 503-522 | - |
| swPAf1 | ATGGAAGACTTTGTGCGACA | 1-20 | + |
| swPAf2 | ACACCCTTGACGAAGAGAGC | 482-501 | + |
| swPAf3 | AATTACCTCATGGCTTGGAA | 997-1016 | + |
| swPAf4 | CTGACCCGAGACTGGAGCCA | 1583-1602 | + |
| swPAr1 | CTACTTCAGTGCATGTGTGA | 2132-2151 | - |
| swPAr2 | GTCCTCAAGAGCATGTCTCC | 1636-1655 | - |
| swPAr3 | GAGTGCCCACTTCAATTGGC | 1091-1110 | - |
| swPAr4 | GGAATCCCATAGACTCCTAC | 551-570 | - |
| swPB1f1 | ATGGATGTCAATCCGACTCT | 1-20 | + |
| swPB1f2 | GGATGTAATGGAATCAATGA | 504-523 | + |
| swPB1f3 | CACCCATAATGTTCTCAAAC | 1019-1038 | + |
| swPB1f4 | TGACCTTGGACCTGCAACGG | 1611-1630 | + |
| swPB1r1 | TTATTTTTGCCGTCTGAGTT | 2255-2274 | - |
| swPB1r2 | CCCTATGGCACCTATATGTG | 1671-1690 | - |
| swPB1r3 | TCTTCATTCTTTTACTCTCG | 1071-1090 | - |
| swPB1r4 | GTCATGTTGTCTCTTACTCT | 568-587 | - |
| swPB2f1 | ATGGAGAGAATAAAAGAACT | 1-20 | + |
| swPB2f2 | GAATACTGACATCAGAGTCA | 524-543 | + |
| swPB2f3 | CGGGCAACCTCCAAACACTG | 1037-1056 | + |
| swPB2f4 | CAAGGAACTGAGAAGTTGAC | 1564-1583 | + |
| swPB2r1 | CTAATTGATGGCCATCCGAA | 2261-2280 | - |
| swPB2r2 | TTGACTAGCACTGACTCAGG | 1624-1643 | - |
| swPB2r3 | TGCCTTTCTGAGAATAGCTG | 1112-1131 | - |
| swPB2r4 | ACGCCACCATCAAGGGAGC | 595-613 | - |

^a^ Sequence coordinates are relative to the coding sequences of the genome segments; not the full length virus segment.
