# Supplementary material for: Nationwide Molecular Surveillance of Pandemic H1N1 Influenza A Virus Genomes: Canada, 2009
Source: PLoS One. 2011 Jan 7;6(1):e16087. doi: 10.1371/journal.pone.0016087 (PMC3017559; doi:10.1371/journal.pone.0016087)
Supplement: Table S7 — Accession numbers for Influenza A/H1N1pdm viruses sequenced in this study. (DOC) [file pone.0016087.s009.doc]

**Table S7.** Accession numbers for Influenza A/H1N1pdm viruses sequenced in this study

| **#** | **Full Accession Name** | **Short Name** | **PB2** | **PB1** | **PA** | **HA** | **NP** | **NA** | **MP** | **NS** |
| --- | --- | --- | --- | --- | --- | --- | --- | --- | --- | --- |
| 1 | A/Canada-NB/RV0005-10/2009(H1N1) | RV0005-10/2009 | HQ240863 | HQ240656 | HQ240449 | HQ239414 | HQ240035 | HQ239828 | HQ239621 | HQ240242 |
| 2 | A/Canada-MB/RV0062-10/2009(H1N1) | RV0062-10/2009 | HQ240864 | HQ240657 | HQ240450 | HQ239415 | HQ240036 | HQ239829 | HQ239622 | HQ240243 |
| 3 | A/Canada-ON/RV1526/2009(H1N1) | RV1526/2009 | GQ132137 | GQ132180 | GQ132177 | GQ132143 | GQ132162 | GQ132159 | GQ132153 | GQ132171 |
| 4 | A/Canada-ON/RV1527/2009(H1N1) | RV1527/2009 | FJ998205 | FJ998227 | FJ998224 | FJ998209 | FJ998218 | FJ998215 | FJ998212 | FJ998221 |
| 5 | A/Canada-ON/RV1529/2009(H1N1) | RV1529/2009 | GQ132139 | GQ132181 | GQ132173 | GQ132144 | GQ132163 | GQ132157 | GQ132149 | GQ132168 |
| 6 | A/Canada-AB/RV1531/2009(H1N1) | RV1531/2009 | GQ465661 | GQ465745 | GQ465733 | GQ465673 | GQ465709 | GQ465697 | GQ465685 | GQ465721 |
| 7 | A/Canada-AB/RV1532/2009(H1N1) | RV1532/2009 | GQ132140 | GQ132182 | GQ132174 | GQ132146 | GQ132164 | GQ132156 | GQ132151 | GQ132169 |
| 8 | A/Canada-NS/RV1535/2009(H1N1) | RV1535/2009 | GQ120442 | FJ998225 | FJ998222 | FJ998207 | FJ998216 | FJ998213 | FJ998210 | FJ998219 |
| 9 | A/Canada-NS/RV1536/2009(H1N1) | RV1536/2009 | GQ132141 | GQ132183 | GQ132175 | GQ132147 | GQ132165 | GQ132158 | GQ132152 | GQ132170 |
| 10 | A/Canada-NS/RV1538/2009(H1N1) | RV1538/2009 | GQ132136 | GQ132178 | GQ132172 | GQ132142 | GQ132160 | GQ132154 | GQ132148 | GQ132166 |
| 11 | A/Canada-ON/RV1545/2009(H1N1) | RV1545/2009 | HQ240865 | HQ240658 | HQ240451 | HQ239416 | HQ240037 | HQ239830 | HQ239623 | HQ240244 |
| 12 | A/Canada-NB/RV1546/2009(H1N1) | RV1546/2009 | HQ240866 | HQ240659 | HQ240452 | HQ239417 | HQ240038 | HQ239831 | HQ239624 | HQ240245 |
| 13 | A/Canada-NS/RV1551/2009(H1N1) | RV1551/2009 | GQ465662 | GQ465746 | GQ465734 | GQ465674 | GQ465710 | GQ465698 | GQ465686 | GQ465722 |
| 14 | A/Canada-NS/RV1552/2009(H1N1) | RV1552/2009 | HQ240867 | HQ240660 | HQ240453 | HQ239418 | HQ240039 | HQ239832 | HQ239625 | HQ240246 |
| 15 | A/Canada-NS/RV1554/2009(H1N1) | RV1554/2009 | GQ465663 | GQ465747 | GQ465735 | GQ465675 | GQ465711 | GQ465699 | GQ465687 | GQ465723 |
| 16 | A/Canada-NS/RV1559/2009(H1N1) | RV1559/2009 | HQ240868 | HQ240661 | HQ240454 | HQ239419 | HQ240040 | HQ239833 | HQ239626 | GQ402279 |
| 17 | A/Canada-NS/RV1560/2009(H1N1) | RV1560/2009 | HQ240869 | HQ240662 | HQ240455 | HQ239420 | HQ240041 | HQ239834 | HQ239627 | HQ240247 |
| 18 | A/Canada-NS/RV1561/2009(H1N1) | RV1561/2009 | HQ240870 | HQ240663 | HQ240456 | HQ239421 | HQ240042 | HQ239835 | HQ239628 | HQ240248 |
| 19 | A/Canada-NS/RV1562/2009(H1N1) | RV1562/2009 | HQ240871 | HQ240664 | HQ240457 | HQ239422 | HQ240043 | HQ239836 | HQ239629 | HQ240249 |
| 20 | A/Canada-NS/RV1565/2009(H1N1) | RV1565/2009 | GQ465664 | GQ465748 | GQ465736 | GQ465676 | GQ465712 | GQ465707 | GQ465688 | HQ240250 |
| 21 | A/Canada-NS/RV1572/2009(H1N1) | RV1572/2009 | HQ240872 | HQ240665 | HQ240458 | HQ239423 | HQ240044 | HQ239837 | HQ239630 | GQ465724 |
| 22 | A/Canada-PQ/RV1582/2009(H1N1) | RV1582/2009 | HQ240873 | HQ240666 | HQ240459 | HQ239424 | HQ240045 | HQ239838 | HQ239631 | HQ240251 |
| 23 | A/Canada-ON/RV1586/2009(H1N1) | RV1586/2009 | GQ465665 | GQ465749 | GQ465737 | GQ465677 | GQ465713 | GQ465700 | GQ465689 | HQ240252 |
| 24 | A/Canada-ON/RV1589/2009(H1N1) | RV1589/2009 | GQ465666 | GQ465750 | GQ465738 | GQ465678 | GQ465714 | GQ465701 | GQ465690 | GQ465725 |
| 25 | A/Canada-PQ/RV1590/2009(H1N1) | RV1590/2009 | HQ240874 | HQ240667 | HQ240460 | HQ239425 | HQ240046 | HQ239839 | HQ239632 | GQ465726 |
| 26 | A/Canada-PQ/RV1595/2009(H1N1) | RV1595/2009 | GQ402174 | GQ402314 | GQ402294 | GQ402194 | GQ402254 | GQ402234 | GQ402214 | HQ240253 |
| 27 | A/Canada-AB/RV1602/2009(H1N1) | RV1602/2009 | HQ240875 | HQ240668 | HQ240461 | HQ239426 | HQ240047 | HQ239840 | HQ239633 | GQ402274 |
| 28 | A/Canada-SK/RV1644/2009(H1N1) | RV1644/2009 | GQ465667 | GQ465751 | GQ465739 | GQ465679 | GQ465715 | GQ465702 | GQ465691 | HQ240254 |
| 29 | A/Canada-BC/RV1683/2009(H1N1) | RV1683/2009 | HQ240876 | HQ240669 | HQ240462 | HQ239427 | HQ240048 | HQ239841 | HQ239634 | GQ465727 |
| 30 | A/Canada-BC/RV1718/2009(H1N1) | RV1718/2009 | HQ240877 | HQ240670 | HQ240463 | HQ239428 | HQ240049 | HQ239842 | HQ239635 | HQ240255 |
| 31 | A/Canada-BC/RV1719/2009(H1N1) | RV1719/2009 | HQ240878 | HQ240671 | HQ240464 | HQ239429 | HQ240050 | HQ239843 | HQ239636 | HQ240256 |
| 32 | A/Canada-PQ/RV1720/2009(H1N1) | RV1720/2009 | HQ240879 | HQ240672 | HQ240465 | HQ239430 | HQ240051 | HQ239844 | HQ239637 | HQ240257 |
| 33 | A/Canada-PQ/RV1758/2009(H1N1) | RV1758/2009 | GQ465668 | GQ465752 | GQ465740 | GQ465680 | GQ465716 | GQ465703 | GQ465692 | HQ240258 |
| 34 | A/Canada-MB/RV1759/2009(H1N1) | RV1759/2009 | GQ402175 | GQ402315 | GQ402295 | GQ402195 | GQ402255 | GQ402235 | GQ402215 | GQ465728 |
| 35 | A/Canada-PQ/RV1765/2009(H1N1) | RV1765/2009 | HQ240880 | HQ240673 | HQ240466 | HQ239431 | HQ240052 | HQ239845 | HQ239638 | GQ402275 |
| 36 | A/Canada-SK/RV1767/2009(H1N1) | RV1767/2009 | GQ402176 | GQ402316 | GQ402296 | GQ402196 | GQ402256 | GQ402236 | GQ402216 | HQ240259 |
| 37 | A/Canada-SK/RV1788/2009(H1N1) | RV1788/2009 | GQ465669 | GQ465753 | GQ465741 | GQ465681 | GQ465717 | GQ465704 | GQ465693 | GQ402276 |
| 38 | A/Canada-SK/RV1793/2009(H1N1) | RV1793/2009 | GQ402177 | GQ402317 | GQ402297 | GQ402197 | GQ402257 | GQ402237 | GQ402217 | GQ465729 |
| 39 | A/Canada-SK/RV1794/2009(H1N1) | RV1794/2009 | GQ465670 | GQ465754 | GQ465742 | GQ465682 | GQ465718 | GQ465705 | GQ465694 | GQ402277 |
| 40 | A/Canada-SK/RV1797/2009(H1N1) | RV1797/2009 | GQ465671 | GQ465755 | GQ465743 | GQ465683 | GQ465719 | GQ465706 | GQ465695 | GQ465730 |
| 41 | A/Canada-MB/RV1798/2009(H1N1) | RV1798/2009 | GQ402178 | GQ402318 | GQ402298 | GQ402198 | GQ402258 | GQ402238 | GQ402218 | GQ465731 |
| 42 | A/Canada-MB/RV1923/2009(H1N1) | RV1923/2009 | HQ240881 | HQ240674 | HQ240467 | HQ239432 | HQ240053 | HQ239846 | HQ239639 | GQ402278 |
| 43 | A/Canada-PQ/RV1927/2009(H1N1) | RV1927/2009 | HQ240882 | HQ240675 | HQ240468 | HQ239433 | HQ240054 | HQ239847 | HQ239640 | HQ240260 |
| 44 | A/Canada-PQ/RV1954/2009(H1N1) | RV1954/2009 | GQ402179 | GQ402319 | GQ402299 | GQ402199 | GQ402259 | GQ402239 | GQ402219 | HQ240261 |
| 45 | A/Canada-MB/RV1964/2009(H1N1) | RV1964/2009 | GQ402180 | GQ402320 | GQ402300 | GQ402200 | GQ402260 | GQ402240 | GQ402220 | GQ402280 |
| 46 | A/Canada-MB/RV1967/2009(H1N1) | RV1967/2009 | HQ240883 | HQ240676 | HQ240469 | HQ239434 | HQ240055 | HQ239848 | HQ239641 | HQ240262 |
| 47 | A/Canada-MB/RV1968/2009(H1N1) | RV1968/2009 | HQ240884 | HQ240677 | HQ240470 | HQ239435 | HQ240056 | HQ239849 | HQ239642 | HQ240263 |
| 48 | A/Canada-MB/RV1971/2009(H1N1) | RV1971/2009 | HQ240885 | HQ240678 | HQ240471 | HQ239436 | HQ240057 | HQ239850 | HQ239643 | HQ240264 |
| 49 | A/Canada-MB/RV1973/2009(H1N1) | RV1973/2009 | HQ240886 | HQ240679 | HQ240472 | HQ239437 | HQ240058 | HQ239851 | HQ239644 | HQ240265 |
| 50 | A/Canada-MB/RV1975/2009(H1N1) | RV1975/2009 | GQ402181 | GQ402321 | GQ402301 | GQ402201 | GQ402261 | GQ402241 | GQ402221 | GQ402281 |
| 51 | A/Canada-MB/RV1976/2009(H1N1) | RV1976/2009 | HQ240887 | HQ240680 | HQ240473 | HQ239438 | HQ240059 | HQ239852 | HQ239645 | HQ240266 |
| 52 | A/Canada-MB/RV1977/2009(H1N1) | RV1977/2009 | GQ402182 | GQ402322 | GQ402302 | GQ402202 | GQ402262 | GQ402242 | GQ402222 | GQ402282 |
| 53 | A/Canada-MB/RV1982/2009(H1N1) | RV1982/2009 | GQ402183 | GQ402323 | GQ402303 | GQ402203 | GQ402263 | GQ402243 | GQ402223 | GQ402283 |
| 54 | A/Canada-MB/RV1983/2009(H1N1) | RV1983/2009 | HQ240888 | HQ240681 | HQ240474 | HQ239439 | HQ240060 | HQ239853 | HQ239646 | HQ240267 |
| 55 | A/Canada-MB/RV1985/2009(H1N1) | RV1985/2009 | HQ240889 | HQ240682 | HQ240475 | HQ239440 | HQ240061 | HQ239854 | HQ239647 | HQ240268 |
| 56 | A/Canada-MB/RV1989/2009(H1N1) | RV1989/2009 | HQ240890 | HQ240683 | HQ240476 | HQ239441 | HQ240062 | HQ239855 | HQ239648 | HQ240269 |
| 57 | A/Canada-MB/RV2001/2009(H1N1) | RV2001/2009 | HQ240891 | HQ240684 | HQ240477 | HQ239442 | HQ240063 | HQ239856 | HQ239649 | HQ240270 |
| 58 | A/Canada-MB/RV2003/2009(H1N1) | RV2003/2009 | HQ240892 | HQ240685 | HQ240478 | HQ239443 | HQ240064 | HQ239857 | HQ239650 | HQ240271 |
| 59 | A/Canada-MB/RV2003-10/2009(H1N1) | RV2003-10/2009 | HQ240893 | HQ240686 | HQ240479 | HQ239444 | HQ240065 | HQ239858 | HQ239651 | HQ240272 |
| 60 | A/Canada-MB/RV2004/2009(H1N1) | RV2004/2009 | HQ240894 | HQ240687 | HQ240480 | HQ239445 | HQ240066 | HQ239859 | HQ239652 | HQ240273 |
| 61 | A/Canada-BC/RV2004-10/2009(H1N1) | RV2004-10/2009 | HQ240895 | HQ240688 | HQ240481 | HQ239446 | HQ240067 | HQ239860 | HQ239653 | HQ240274 |
| 62 | A/Canada-MB/RV2005/2009(H1N1) | RV2005/2009 | HQ240896 | HQ240689 | HQ240482 | HQ239447 | HQ240068 | HQ239861 | HQ239654 | HQ240275 |
| 63 | A/Canada-BC/RV2005-10/2009(H1N1) | RV2005-10/2009 | HQ240897 | HQ240690 | HQ240483 | HQ239448 | HQ240069 | HQ239862 | HQ239655 | HQ240276 |
| 64 | A/Canada-MB/RV2006-10/2009(H1N1) | RV2006-10/2009 | HQ240898 | HQ240691 | HQ240484 | HQ239449 | HQ240070 | HQ239863 | HQ239656 | HQ240277 |
| 65 | A/Canada-MB/RV2007-10/2009(H1N1) | RV2007-10/2009 | HQ240899 | HQ240692 | HQ240485 | HQ239450 | HQ240071 | HQ239864 | HQ239657 | HQ240278 |
| 66 | A/Canada-MB/RV2010/2009(H1N1) | RV2010/2009 | HQ240900 | HQ240693 | HQ240486 | HQ239451 | HQ240072 | HQ239865 | HQ239658 | HQ240279 |
| 67 | A/Canada-MB/RV2010-10/2009(H1N1) | RV2010-10/2009 | HQ240901 | HQ240694 | HQ240487 | HQ239452 | HQ240073 | HQ239866 | HQ239659 | HQ240280 |
| 68 | A/Canada-MB/RV2011-10/2009(H1N1) | RV2011-10/2009 | HQ240902 | HQ240695 | HQ240488 | HQ239453 | HQ240074 | HQ239867 | HQ239660 | HQ240281 |
| 69 | A/Canada-MB/RV2012-10/2009(H1N1) | RV2012-10/2009 | HQ240903 | HQ240696 | HQ240489 | HQ239454 | HQ240075 | HQ239868 | HQ239661 | HQ240282 |
| 70 | A/Canada-MB/RV2013/2009(H1N1) | RV2013/2009 | GQ402184 | GQ402324 | GQ402304 | GQ402204 | GQ402264 | GQ402244 | GQ402224 | GQ402284 |
| 71 | A/Canada-MB/RV2013-10/2009(H1N1) | RV2013-10/2009 | HQ240904 | HQ240697 | HQ240490 | HQ239455 | HQ240076 | HQ239869 | HQ239662 | HQ240283 |
| 72 | A/Canada-MB/RV2014-10/2009(H1N1) | RV2014-10/2009 | HQ240905 | HQ240698 | HQ240491 | HQ239456 | HQ240077 | HQ239870 | HQ239663 | HQ240284 |
| 73 | A/Canada-MB/RV2015/2009(H1N1) | RV2015/2009 | HQ240906 | HQ240699 | HQ240492 | HQ239457 | HQ240078 | HQ239871 | HQ239664 | HQ240285 |
| 74 | A/Canada-ON/RV2015-10/2009(H1N1) | RV2015-10/2009 | HQ240907 | HQ240700 | HQ240493 | HQ239458 | HQ240079 | HQ239872 | HQ239665 | HQ240286 |
| 75 | A/Canada-ON/RV2016-10/2009(H1N1) | RV2016-10/2009 | HQ240908 | HQ240701 | HQ240494 | HQ239459 | HQ240080 | HQ239873 | HQ239666 | HQ240287 |
| 76 | A/Canada-ON/RV2017-10/2009(H1N1) | RV2017-10/2009 | HQ240909 | HQ240702 | HQ240495 | HQ239460 | HQ240081 | HQ239874 | HQ239667 | HQ240288 |
| 77 | A/Canada-MB/RV2018/2009(H1N1) | RV2018/2009 | GQ402185 | GQ402325 | GQ402305 | GQ402205 | GQ402265 | GQ402245 | GQ402225 | GQ402285 |
| 78 | A/Canada-MB/RV2018-10/2009(H1N1) | RV2018-10/2009 | HQ240910 | HQ240703 | HQ240496 | HQ239461 | HQ240082 | HQ239875 | HQ239668 | HQ240289 |
| 79 | A/Canada-MB/RV2020/2009(H1N1) | RV2020/2009 | GQ402186 | GQ402326 | GQ402306 | GQ402206 | GQ402266 | GQ402246 | GQ402226 | GQ402286 |
| 80 | A/Canada-MB/RV2023/2009(H1N1) | RV2023/2009 | GQ402167 | GQ402307 | GQ402287 | GQ402187 | GQ402247 | GQ402227 | GQ402207 | GQ402267 |
| 81 | A/Canada-MB/RV2026-10/2009(H1N1) | RV2026-10/2009 | HQ240911 | HQ240704 | HQ240497 | HQ239462 | HQ240083 | HQ239876 | HQ239669 | HQ240290 |
| 82 | A/Canada-MB/RV2027-10/2009(H1N1) | RV2027-10/2009 | HQ240912 | HQ240705 | HQ240498 | HQ239463 | HQ240084 | HQ239877 | HQ239670 | HQ240291 |
| 83 | A/Canada-MB/RV2028-10/2009(H1N1) | RV2028-10/2009 | HQ240913 | HQ240706 | HQ240499 | HQ239464 | HQ240085 | HQ239878 | HQ239671 | HQ240292 |
| 84 | A/Canada-MB/RV2029-10/2009(H1N1) | RV2029-10/2009 | HQ240914 | HQ240707 | HQ240500 | HQ239465 | HQ240086 | HQ239879 | HQ239672 | HQ240293 |
| 85 | A/Canada-MB/RV2030-10/2009(H1N1) | RV2030-10/2009 | HQ240915 | HQ240708 | HQ240501 | HQ239466 | HQ240087 | HQ239880 | HQ239673 | HQ240294 |
| 86 | A/Canada-MB/RV2031-10/2009(H1N1) | RV2031-10/2009 | HQ240916 | HQ240709 | HQ240502 | HQ239467 | HQ240088 | HQ239881 | HQ239674 | HQ240295 |
| 87 | A/Canada-MB/RV2032-10/2009(H1N1) | RV2032-10/2009 | HQ240917 | HQ240710 | HQ240503 | HQ239468 | HQ240089 | HQ239882 | HQ239675 | HQ240296 |
| 88 | A/Canada-MB/RV2033-10/2009(H1N1) | RV2033-10/2009 | HQ240918 | HQ240711 | HQ240504 | HQ239469 | HQ240090 | HQ239883 | HQ239676 | HQ240297 |
| 89 | A/Canada-MB/RV2034-10/2009(H1N1) | RV2034-10/2009 | HQ240919 | HQ240712 | HQ240505 | HQ239470 | HQ240091 | HQ239884 | HQ239677 | HQ240298 |
| 90 | A/Canada-MB/RV2036-10/2009(H1N1) | RV2036-10/2009 | HQ240920 | HQ240713 | HQ240506 | HQ239471 | HQ240092 | HQ239885 | HQ239678 | HQ240299 |
| 91 | A/Canada-MB/RV2037-10/2009(H1N1) | RV2037-10/2009 | HQ240921 | HQ240714 | HQ240507 | HQ239472 | HQ240093 | HQ239886 | HQ239679 | HQ240300 |
| 92 | A/Canada-MB/RV2038-10/2009(H1N1) | RV2038-10/2009 | HQ240922 | HQ240715 | HQ240508 | HQ239473 | HQ240094 | HQ239887 | HQ239680 | HQ240301 |
| 93 | A/Canada-MB/RV2039-10/2009(H1N1) | RV2039-10/2009 | HQ240923 | HQ240716 | HQ240509 | HQ239474 | HQ240095 | HQ239888 | HQ239681 | HQ240302 |
| 94 | A/Canada-MB/RV2040/2009(H1N1) | RV2040/2009 | HQ240924 | HQ240717 | HQ240510 | HQ239475 | HQ240096 | HQ239889 | HQ239682 | HQ240303 |
| 95 | A/Canada-MB/RV2040-10/2009(H1N1) | RV2040-10/2009 | HQ240925 | HQ240718 | HQ240511 | HQ239476 | HQ240097 | HQ239890 | HQ239683 | HQ240304 |
| 96 | A/Canada-MB/RV2041/2009(H1N1) | RV2041/2009 | HQ240926 | HQ240719 | HQ240512 | HQ239477 | HQ240098 | HQ239891 | HQ239684 | HQ240305 |
| 97 | A/Canada-MB/RV2041-10/2009(H1N1) | RV2041-10/2009 | HQ240927 | HQ240720 | HQ240513 | HQ239478 | HQ240099 | HQ239892 | HQ239685 | HQ240306 |
| 98 | A/Canada-MB/RV2042-10/2009(H1N1) | RV2042-10/2009 | HQ240930 | HQ240723 | HQ240516 | HQ239481 | HQ240102 | HQ239895 | HQ239688 | HQ240309 |
| 99 | A/Canada-MB/RV2042i/2009(H1N1) | RV2042i/2009 | HQ240928 | HQ240721 | HQ240514 | HQ239479 | HQ240100 | HQ239893 | HQ239686 | HQ240307 |
| 100 | A/Canada-MB/RV2042p/2009(H1N1) | RV2042p/2009 | HQ240929 | HQ240722 | HQ240515 | HQ239480 | HQ240101 | HQ239894 | HQ239687 | HQ240308 |
| 101 | A/Canada-MB/RV2043/2009(H1N1) | RV2043/2009 | HQ240931 | HQ240724 | HQ240517 | HQ239482 | HQ240103 | HQ239896 | HQ239689 | HQ240310 |
| 102 | A/Canada-MB/RV2043-10/2009(H1N1) | RV2043-10/2009 | HQ240932 | HQ240725 | HQ240518 | HQ239483 | HQ240104 | HQ239897 | HQ239690 | HQ240311 |
| 103 | A/Canada-MB/RV2044/2009(H1N1) | RV2044/2009 | HQ240933 | HQ240726 | HQ240519 | HQ239484 | HQ240105 | HQ239898 | HQ239691 | HQ240312 |
| 104 | A/Canada-MB/RV2044-10/2009(H1N1) | RV2044-10/2009 | HQ240934 | HQ240727 | HQ240520 | HQ239485 | HQ240106 | HQ239899 | HQ239692 | HQ240313 |
| 105 | A/Canada-MB/RV2045-10/2009(H1N1) | RV2045-10/2009 | HQ240935 | HQ240728 | HQ240521 | HQ239486 | HQ240107 | HQ239900 | HQ239693 | HQ240314 |
| 106 | A/Canada-MB/RV2046/2009(H1N1) | RV2046/2009 | HQ240936 | HQ240729 | HQ240522 | HQ239487 | HQ240108 | HQ239901 | HQ239694 | HQ240315 |
| 107 | A/Canada-MB/RV2046-10/2009(H1N1) | RV2046-10/2009 | HQ240937 | HQ240730 | HQ240523 | HQ239488 | HQ240109 | HQ239902 | HQ239695 | HQ240316 |
| 108 | A/Canada-MB/RV2049/2009(H1N1) | RV2049/2009 | HQ240938 | HQ240731 | HQ240524 | HQ239489 | HQ240110 | HQ239903 | HQ239696 | HQ240317 |
| 109 | A/Canada-MB/RV2051/2009(H1N1) | RV2051/2009 | HQ240939 | HQ240732 | HQ240525 | HQ239490 | HQ240111 | HQ239904 | HQ239697 | HQ240318 |
| 110 | A/Canada-MB/RV2053/2009(H1N1) | RV2053/2009 | HQ240940 | HQ240733 | HQ240526 | HQ239491 | HQ240112 | HQ239905 | HQ239698 | HQ240319 |
| 111 | A/Canada-MB/RV2054/2009(H1N1) | RV2054/2009 | HQ240941 | HQ240734 | HQ240527 | HQ239492 | HQ240113 | HQ239906 | HQ239699 | HQ240320 |
| 112 | A/Canada-MB/RV2055/2009(H1N1) | RV2055/2009 | HQ240942 | HQ240735 | HQ240528 | HQ239493 | HQ240114 | HQ239907 | HQ239700 | HQ240321 |
| 113 | A/Canada-MB/RV2058/2009(H1N1) | RV2058/2009 | HQ240943 | HQ240736 | HQ240529 | HQ239494 | HQ240115 | HQ239908 | HQ239701 | HQ240322 |
| 114 | A/Canada-MB/RV2060/2009(H1N1) | RV2060/2009 | HQ240944 | HQ240737 | HQ240530 | HQ239495 | HQ240116 | HQ239909 | HQ239702 | HQ240323 |
| 115 | A/Canada-MB/RV2063/2009(H1N1) | RV2063/2009 | HQ240945 | HQ240738 | HQ240531 | HQ239496 | HQ240117 | HQ239910 | HQ239703 | HQ240324 |
| 116 | A/Canada-MB/RV2064/2009(H1N1) | RV2064/2009 | HQ240946 | HQ240739 | HQ240532 | HQ239497 | HQ240118 | HQ239911 | HQ239704 | HQ240325 |
| 117 | A/Canada-MB/RV2066i/2009(H1N1) | RV2066i/2009 | HQ240947 | HQ240740 | HQ240533 | HQ239498 | HQ240119 | HQ239912 | HQ239705 | HQ240326 |
| 118 | A/Canada-MB/RV2066p/2009(H1N1) | RV2066p/2009 | HQ240948 | HQ240741 | HQ240534 | HQ239499 | HQ240120 | HQ239913 | HQ239706 | HQ240327 |
| 119 | A/Canada-MB/RV2067/2009(H1N1) | RV2067/2009 | HQ240949 | HQ240742 | HQ240535 | HQ239500 | HQ240121 | HQ239914 | HQ239707 | HQ240328 |
| 120 | A/Canada-MB/RV2069/2009(H1N1) | RV2069/2009 | HQ240950 | HQ240743 | HQ240536 | HQ239501 | HQ240122 | HQ239915 | HQ239708 | HQ240329 |
| 121 | A/Canada-MB/RV2076i/2009(H1N1) | RV2076i/2009 | HQ240951 | HQ240744 | HQ240537 | HQ239502 | HQ240123 | HQ239916 | HQ239709 | HQ240330 |
| 122 | A/Canada-MB/RV2076p/2009(H1N1) | RV2076p/2009 | HQ240952 | HQ240745 | HQ240538 | HQ239503 | HQ240124 | HQ239917 | HQ239710 | HQ240331 |
| 123 | A/Canada-MB/RV2103/2009(H1N1) | RV2103/2009 | HQ240953 | HQ240746 | HQ240539 | HQ239504 | HQ240125 | HQ239918 | HQ239711 | HQ240332 |
| 124 | A/Canada-MB/RV2105/2009(H1N1) | RV2105/2009 | HQ240954 | HQ240747 | HQ240540 | HQ239505 | HQ240126 | HQ239919 | HQ239712 | HQ240333 |
| 125 | A/Canada-MB/RV2107/2009(H1N1) | RV2107/2009 | HQ240955 | HQ240748 | HQ240541 | HQ239506 | HQ240127 | HQ239920 | HQ239713 | HQ240334 |
| 126 | A/Canada-MB/RV2110/2009(H1N1) | RV2110/2009 | HQ240956 | HQ240749 | HQ240542 | HQ239507 | HQ240128 | HQ239921 | HQ239714 | HQ240335 |
| 127 | A/Canada-MB/RV2121/2009(H1N1) | RV2121/2009 | HQ240957 | HQ240750 | HQ240543 | HQ239508 | HQ240129 | HQ239922 | HQ239715 | HQ240336 |
| 128 | A/Canada-MB/RV2123/2009(H1N1) | RV2123/2009 | HQ240958 | HQ240751 | HQ240544 | HQ239509 | HQ240130 | HQ239923 | HQ239716 | HQ240337 |
| 129 | A/Canada-MB/RV2132/2009(H1N1) | RV2132/2009 | HQ240959 | HQ240752 | HQ240545 | HQ239510 | HQ240131 | HQ239924 | HQ239717 | HQ240338 |
| 130 | A/Canada-MB/RV2138/2009(H1N1) | RV2138/2009 | HQ240960 | HQ240753 | HQ240546 | HQ239511 | HQ240132 | HQ239925 | HQ239718 | HQ240339 |
| 131 | A/Canada-MB/RV2146/2009(H1N1) | RV2146/2009 | HQ240961 | HQ240754 | HQ240547 | HQ239512 | HQ240133 | HQ239926 | HQ239719 | HQ240340 |
| 132 | A/Canada-MB/RV2147/2009(H1N1) | RV2147/2009 | HQ240962 | HQ240755 | HQ240548 | HQ239513 | HQ240134 | HQ239927 | HQ239720 | HQ240341 |
| 133 | A/Canada-MB/RV2152/2009(H1N1) | RV2152/2009 | HQ240963 | HQ240756 | HQ240549 | HQ239514 | HQ240135 | HQ239928 | HQ239721 | HQ240342 |
| 134 | A/Canada-NB/RV2276/2009(H1N1) | RV2276/2009 | HQ240964 | HQ240757 | HQ240550 | HQ239515 | HQ240136 | HQ239929 | HQ239722 | HQ240343 |
| 135 | A/Canada-MB/RV2291/2009(H1N1) | RV2291/2009 | HQ240965 | HQ240758 | HQ240551 | HQ239516 | HQ240137 | HQ239930 | HQ239723 | HQ240344 |
| 136 | A/Canada-NB/RV2431/2009(H1N1) | RV2431/2009 | HQ240966 | HQ240759 | HQ240552 | HQ239517 | HQ240138 | HQ239931 | HQ239724 | HQ240345 |
| 137 | A/Canada-NB/RV2456/2009(H1N1) | RV2456/2009 | HQ240967 | HQ240760 | HQ240553 | HQ239518 | HQ240139 | HQ239932 | HQ239725 | HQ240346 |
| 138 | A/Canada-NB/RV2457/2009(H1N1) | RV2457/2009 | HQ240968 | HQ240761 | HQ240554 | HQ239519 | HQ240140 | HQ239933 | HQ239726 | HQ240347 |
| 139 | A/Canada-NB/RV2458/2009(H1N1) | RV2458/2009 | HQ240969 | HQ240762 | HQ240555 | HQ239520 | HQ240141 | HQ239934 | HQ239727 | HQ240348 |
| 140 | A/Canada-NB/RV2474/2009(H1N1) | RV2474/2009 | HQ240970 | HQ240763 | HQ240556 | HQ239521 | HQ240142 | HQ239935 | HQ239728 | HQ240349 |
| 141 | A/Canada-NB/RV2475/2009(H1N1) | RV2475/2009 | HQ240971 | HQ240764 | HQ240557 | HQ239522 | HQ240143 | HQ239936 | HQ239729 | HQ240350 |
| 142 | A/Canada-NB/RV2476/2009(H1N1) | RV2476/2009 | HQ240972 | HQ240765 | HQ240558 | HQ239523 | HQ240144 | HQ239937 | HQ239730 | HQ240351 |
| 143 | A/Canada-NB/RV2478/2009(H1N1) | RV2478/2009 | HQ240973 | HQ240766 | HQ240559 | HQ239524 | HQ240145 | HQ239938 | HQ239731 | HQ240352 |
| 144 | A/Canada-NB/RV2479/2009(H1N1) | RV2479/2009 | HQ240974 | HQ240767 | HQ240560 | HQ239525 | HQ240146 | HQ239939 | HQ239732 | HQ240353 |
| 145 | A/Canada-SK/RV2486/2009(H1N1) | RV2486/2009 | GQ465660 | GQ465744 | GQ465732 | GQ465672 | GQ465708 | GQ465696 | GQ465684 | GQ465720 |
| 146 | A/Canada-NB/RV2488/2009(H1N1) | RV2488/2009 | HQ240975 | HQ240768 | HQ240561 | HQ239526 | HQ240147 | HQ239940 | HQ239733 | HQ240354 |
| 147 | A/Canada-NB/RV2490/2009(H1N1) | RV2490/2009 | HQ240976 | HQ240769 | HQ240562 | HQ239527 | HQ240148 | HQ239941 | HQ239734 | HQ240355 |
| 148 | A/Canada-NFL/RV2502/2009(H1N1) | RV2502/2009 | HQ240977 | HQ240770 | HQ240563 | HQ239528 | HQ240149 | HQ239942 | HQ239735 | HQ240356 |
| 149 | A/Canada-NFL/RV2503/2009(H1N1) | RV2503/2009 | HQ240978 | HQ240771 | HQ240564 | HQ239529 | HQ240150 | HQ239943 | HQ239736 | HQ240357 |
| 150 | A/Canada-NFL/RV2504/2009(H1N1) | RV2504/2009 | HQ240979 | HQ240772 | HQ240565 | HQ239530 | HQ240151 | HQ239944 | HQ239737 | HQ240358 |
| 151 | A/Canada-NFL/RV2505/2009(H1N1) | RV2505/2009 | HQ240980 | HQ240773 | HQ240566 | HQ239531 | HQ240152 | HQ239945 | HQ239738 | HQ240359 |
| 152 | A/Canada-NFL/RV2506/2009(H1N1) | RV2506/2009 | HQ240981 | HQ240774 | HQ240567 | HQ239532 | HQ240153 | HQ239946 | HQ239739 | HQ240360 |
| 153 | A/Canada-NFL/RV2507/2009(H1N1) | RV2507/2009 | HQ240982 | HQ240775 | HQ240568 | HQ239533 | HQ240154 | HQ239947 | HQ239740 | HQ240361 |
| 154 | A/Canada-NFL/RV2509/2009(H1N1) | RV2509/2009 | HQ240983 | HQ240776 | HQ240569 | HQ239534 | HQ240155 | HQ239948 | HQ239741 | HQ240362 |
| 155 | A/Canada-NFL/RV2510/2009(H1N1) | RV2510/2009 | HQ240984 | HQ240777 | HQ240570 | HQ239535 | HQ240156 | HQ239949 | HQ239742 | HQ240363 |
| 156 | A/Canada-NFL/RV2512/2009(H1N1) | RV2512/2009 | HQ240985 | HQ240778 | HQ240571 | HQ239536 | HQ240157 | HQ239950 | HQ239743 | HQ240364 |
| 157 | A/Canada-NFL/RV2513/2009(H1N1) | RV2513/2009 | HQ240986 | HQ240779 | HQ240572 | HQ239537 | HQ240158 | HQ239951 | HQ239744 | HQ240365 |
| 158 | A/Canada-NS/RV2598/2009(H1N1) | RV2598/2009 | HQ240987 | HQ240780 | HQ240573 | HQ239538 | HQ240159 | HQ239952 | HQ239745 | HQ240366 |
| 159 | A/Canada-NS/RV2599/2009(H1N1) | RV2599/2009 | HQ240988 | HQ240781 | HQ240574 | HQ239539 | HQ240160 | HQ239953 | HQ239746 | HQ240367 |
| 160 | A/Canada-AB/RV2621/2009(H1N1) | RV2621/2009 | HQ240989 | HQ240782 | HQ240575 | HQ239540 | HQ240161 | HQ239954 | HQ239747 | HQ240368 |
| 161 | A/Canada-MB/RV2661/2009(H1N1) | RV2661/2009 | HQ240990 | HQ240783 | HQ240576 | HQ239541 | HQ240162 | HQ239955 | HQ239748 | HQ240369 |
| 162 | A/Canada-AB/RV2735/2009(H1N1) | RV2735/2009 | HQ240991 | HQ240784 | HQ240577 | HQ239542 | HQ240163 | HQ239956 | HQ239749 | HQ240370 |
| 163 | A/Canada-AB/RV2809/2009(H1N1) | RV2809/2009 | HQ240992 | HQ240785 | HQ240578 | HQ239543 | HQ240164 | HQ239957 | HQ239750 | HQ240371 |
| 164 | A/Canada-AB/RV2810/2009(H1N1) | RV2810/2009 | HQ240993 | HQ240786 | HQ240579 | HQ239544 | HQ240165 | HQ239958 | HQ239751 | HQ240372 |
| 165 | A/Canada-AB/RV2811/2009(H1N1) | RV2811/2009 | HQ240994 | HQ240787 | HQ240580 | HQ239545 | HQ240166 | HQ239959 | HQ239752 | HQ240373 |
| 166 | A/Canada-AB/RV2812/2009(H1N1) | RV2812/2009 | HQ240995 | HQ240788 | HQ240581 | HQ239546 | HQ240167 | HQ239960 | HQ239753 | HQ240374 |
| 167 | A/Canada-AB/RV2813/2009(H1N1) | RV2813/2009 | HQ240996 | HQ240789 | HQ240582 | HQ239547 | HQ240168 | HQ239961 | HQ239754 | HQ240375 |
| 168 | A/Canada-AB/RV2814/2009(H1N1) | RV2814/2009 | HQ240997 | HQ240790 | HQ240583 | HQ239548 | HQ240169 | HQ239962 | HQ239755 | HQ240376 |
| 169 | A/Canada-AB/RV2815/2009(H1N1) | RV2815/2009 | HQ240998 | HQ240791 | HQ240584 | HQ239549 | HQ240170 | HQ239963 | HQ239756 | HQ240377 |
| 170 | A/Canada-AB/RV2816/2009(H1N1) | RV2816/2009 | HQ240999 | HQ240792 | HQ240585 | HQ239550 | HQ240171 | HQ239964 | HQ239757 | HQ240378 |
| 171 | A/Canada-AB/RV2817/2009(H1N1) | RV2817/2009 | HQ241000 | HQ240793 | HQ240586 | HQ239551 | HQ240172 | HQ239965 | HQ239758 | HQ240379 |
| 172 | A/Canada-AB/RV2818/2009(H1N1) | RV2818/2009 | HQ241001 | HQ240794 | HQ240587 | HQ239552 | HQ240173 | HQ239966 | HQ239759 | HQ240380 |
| 173 | A/Canada-AB/RV2819/2009(H1N1) | RV2819/2009 | HQ241002 | HQ240795 | HQ240588 | HQ239553 | HQ240174 | HQ239967 | HQ239760 | HQ240381 |
| 174 | A/Canada-AB/RV2820/2009(H1N1) | RV2820/2009 | HQ241003 | HQ240796 | HQ240589 | HQ239554 | HQ240175 | HQ239968 | HQ239761 | HQ240382 |
| 175 | A/Canada-AB/RV2821/2009(H1N1) | RV2821/2009 | HQ241004 | HQ240797 | HQ240590 | HQ239555 | HQ240176 | HQ239969 | HQ239762 | HQ240383 |
| 176 | A/Canada-AB/RV2823/2009(H1N1) | RV2823/2009 | HQ241005 | HQ240798 | HQ240591 | HQ239556 | HQ240177 | HQ239970 | HQ239763 | HQ240384 |
| 177 | A/Canada-AB/RV2824/2009(H1N1) | RV2824/2009 | HQ241006 | HQ240799 | HQ240592 | HQ239557 | HQ240178 | HQ239971 | HQ239764 | HQ240385 |
| 178 | A/Canada-AB/RV2825/2009(H1N1) | RV2825/2009 | HQ241007 | HQ240800 | HQ240593 | HQ239558 | HQ240179 | HQ239972 | HQ239765 | HQ240386 |
| 179 | A/Canada-AB/RV2827/2009(H1N1) | RV2827/2009 | HQ241008 | HQ240801 | HQ240594 | HQ239559 | HQ240180 | HQ239973 | HQ239766 | HQ240387 |
| 180 | A/Canada-AB/RV2828/2009(H1N1) | RV2828/2009 | HQ241009 | HQ240802 | HQ240595 | HQ239560 | HQ240181 | HQ239974 | HQ239767 | HQ240388 |
| 181 | A/Canada-NU/RV2851/2009(H1N1) | RV2851/2009 | HQ241010 | HQ240803 | HQ240596 | HQ239561 | HQ240182 | HQ239975 | HQ239768 | HQ240389 |
| 182 | A/Canada-NWT/RV2852/2009(H1N1) | RV2852/2009 | HQ241011 | HQ240804 | HQ240597 | HQ239562 | HQ240183 | HQ239976 | HQ239769 | HQ240390 |
| 183 | A/Canada-AB/RV2853/2009(H1N1) | RV2853/2009 | HQ241012 | HQ240805 | HQ240598 | HQ239563 | HQ240184 | HQ239977 | HQ239770 | HQ240391 |
| 184 | A/Canada-AB/RV2854/2009(H1N1) | RV2854/2009 | HQ241013 | HQ240806 | HQ240599 | HQ239564 | HQ240185 | HQ239978 | HQ239771 | HQ240392 |
| 185 | A/Canada-NWT/RV2869/2009(H1N1) | RV2869/2009 | HQ241014 | HQ240807 | HQ240600 | HQ239565 | HQ240186 | HQ239979 | HQ239772 | HQ240393 |
| 186 | A/Canada-ON/RV2887/2009(H1N1) | RV2887/2009 | HQ241015 | HQ240808 | HQ240601 | HQ239566 | HQ240187 | HQ239980 | HQ239773 | HQ240394 |
| 187 | A/Canada-ON/RV2888/2009(H1N1) | RV2888/2009 | HQ241016 | HQ240809 | HQ240602 | HQ239567 | HQ240188 | HQ239981 | HQ239774 | HQ240395 |
| 188 | A/Canada-ON/RV2889/2009(H1N1) | RV2889/2009 | HQ241017 | HQ240810 | HQ240603 | HQ239568 | HQ240189 | HQ239982 | HQ239775 | HQ240396 |
| 189 | A/Canada-ON/RV2890/2009(H1N1) | RV2890/2009 | HQ241018 | HQ240811 | HQ240604 | HQ239569 | HQ240190 | HQ239983 | HQ239776 | HQ240397 |
| 190 | A/Canada-ON/RV2891/2009(H1N1) | RV2891/2009 | HQ241019 | HQ240812 | HQ240605 | HQ239570 | HQ240191 | HQ239984 | HQ239777 | HQ240398 |
| 191 | A/Canada-ON/RV2892/2009(H1N1) | RV2892/2009 | HQ241020 | HQ240813 | HQ240606 | HQ239571 | HQ240192 | HQ239985 | HQ239778 | HQ240399 |
| 192 | A/Canada-ON/RV2904/2009(H1N1) | RV2904/2009 | HQ241021 | HQ240814 | HQ240607 | HQ239572 | HQ240193 | HQ239986 | HQ239779 | HQ240400 |
| 193 | A/Canada-ON/RV2905/2009(H1N1) | RV2905/2009 | HQ241022 | HQ240815 | HQ240608 | HQ239573 | HQ240194 | HQ239987 | HQ239780 | HQ240401 |
| 194 | A/Canada-ON/RV2906/2009(H1N1) | RV2906/2009 | HQ241023 | HQ240816 | HQ240609 | HQ239574 | HQ240195 | HQ239988 | HQ239781 | HQ240402 |
| 195 | A/Canada-ON/RV2907/2009(H1N1) | RV2907/2009 | HQ241024 | HQ240817 | HQ240610 | HQ239575 | HQ240196 | HQ239989 | HQ239782 | HQ240403 |
| 196 | A/Canada-ON/RV2908/2009(H1N1) | RV2908/2009 | HQ241025 | HQ240818 | HQ240611 | HQ239576 | HQ240197 | HQ239990 | HQ239783 | HQ240404 |
| 197 | A/Canada-ON/RV2909/2009(H1N1) | RV2909/2009 | HQ241026 | HQ240819 | HQ240612 | HQ239577 | HQ240198 | HQ239991 | HQ239784 | HQ240405 |
| 198 | A/Canada-ON/RV2910/2009(H1N1) | RV2910/2009 | HQ241027 | HQ240820 | HQ240613 | HQ239578 | HQ240199 | HQ239992 | HQ239785 | HQ240406 |
| 199 | A/Canada-ON/RV2912/2009(H1N1) | RV2912/2009 | HQ241028 | HQ240821 | HQ240614 | HQ239579 | HQ240200 | HQ239993 | HQ239786 | HQ240407 |
| 200 | A/Canada-ON/RV2913/2009(H1N1) | RV2913/2009 | HQ241029 | HQ240822 | HQ240615 | HQ239580 | HQ240201 | HQ239994 | HQ239787 | HQ240408 |
| 201 | A/Canada-ON/RV2914/2009(H1N1) | RV2914/2009 | HQ241030 | HQ240823 | HQ240616 | HQ239581 | HQ240202 | HQ239995 | HQ239788 | HQ240409 |
| 202 | A/Canada-SK/RV2924/2009(H1N1) | RV2924/2009 | HQ241031 | HQ240824 | HQ240617 | HQ239582 | HQ240203 | HQ239996 | HQ239789 | HQ240410 |
| 203 | A/Canada-SK/RV2925/2009(H1N1) | RV2925/2009 | HQ241032 | HQ240825 | HQ240618 | HQ239583 | HQ240204 | HQ239997 | HQ239790 | HQ240411 |
| 204 | A/Canada-SK/RV2926/2009(H1N1) | RV2926/2009 | HQ241033 | HQ240826 | HQ240619 | HQ239584 | HQ240205 | HQ239998 | HQ239791 | HQ240412 |
| 205 | A/Canada-SK/RV2927/2009(H1N1) | RV2927/2009 | HQ241034 | HQ240827 | HQ240620 | HQ239585 | HQ240206 | HQ239999 | HQ239792 | HQ240413 |
| 206 | A/Canada-SK/RV2928/2009(H1N1) | RV2928/2009 | HQ241035 | HQ240828 | HQ240621 | HQ239586 | HQ240207 | HQ240000 | HQ239793 | HQ240414 |
| 207 | A/Canada-SK/RV2929/2009(H1N1) | RV2929/2009 | HQ241036 | HQ240829 | HQ240622 | HQ239587 | HQ240208 | HQ240001 | HQ239794 | HQ240415 |
| 208 | A/Canada-SK/RV2930/2009(H1N1) | RV2930/2009 | HQ241037 | HQ240830 | HQ240623 | HQ239588 | HQ240209 | HQ240002 | HQ239795 | HQ240416 |
| 209 | A/Canada-BC/RV2931/2009(H1N1) | RV2931/2009 | HQ241038 | HQ240831 | HQ240624 | HQ239589 | HQ240210 | HQ240003 | HQ239796 | HQ240417 |
| 210 | A/Canada-BC/RV2933/2009(H1N1) | RV2933/2009 | HQ241039 | HQ240832 | HQ240625 | HQ239590 | HQ240211 | HQ240004 | HQ239797 | HQ240418 |
| 211 | A/Canada-BC/RV2934/2009(H1N1) | RV2934/2009 | HQ241040 | HQ240833 | HQ240626 | HQ239591 | HQ240212 | HQ240005 | HQ239798 | HQ240419 |
| 212 | A/Canada-BC/RV2935/2009(H1N1) | RV2935/2009 | HQ241041 | HQ240834 | HQ240627 | HQ239592 | HQ240213 | HQ240006 | HQ239799 | HQ240420 |
| 213 | A/Canada-BC/RV2936/2009(H1N1) | RV2936/2009 | HQ241042 | HQ240835 | HQ240628 | HQ239593 | HQ240214 | HQ240007 | HQ239800 | HQ240421 |
| 214 | A/Canada-BC/RV2937/2009(H1N1) | RV2937/2009 | HQ241043 | HQ240836 | HQ240629 | HQ239594 | HQ240215 | HQ240008 | HQ239801 | HQ240422 |
| 215 | A/Canada-BC/RV2938/2009(H1N1) | RV2938/2009 | HQ241044 | HQ240837 | HQ240630 | HQ239595 | HQ240216 | HQ240009 | HQ239802 | HQ240423 |
| 216 | A/Canada-BC/RV2939/2009(H1N1) | RV2939/2009 | HQ241045 | HQ240838 | HQ240631 | HQ239596 | HQ240217 | HQ240010 | HQ239803 | HQ240424 |
| 217 | A/Canada-BC/RV2940/2009(H1N1) | RV2940/2009 | HQ241046 | HQ240839 | HQ240632 | HQ239597 | HQ240218 | HQ240011 | HQ239804 | HQ240425 |
| 218 | A/Canada-BC/RV2941/2009(H1N1) | RV2941/2009 | HQ241047 | HQ240840 | HQ240633 | HQ239598 | HQ240219 | HQ240012 | HQ239805 | HQ240426 |
| 219 | A/Canada-BC/RV2942/2009(H1N1) | RV2942/2009 | HQ241048 | HQ240841 | HQ240634 | HQ239599 | HQ240220 | HQ240013 | HQ239806 | HQ240427 |
| 220 | A/Canada-BC/RV2943/2009(H1N1) | RV2943/2009 | HQ241049 | HQ240842 | HQ240635 | HQ239600 | HQ240221 | HQ240014 | HQ239807 | HQ240428 |
| 221 | A/Canada-BC/RV2944/2009(H1N1) | RV2944/2009 | HQ241050 | HQ240843 | HQ240636 | HQ239601 | HQ240222 | HQ240015 | HQ239808 | HQ240429 |
| 222 | A/Canada-BC/RV2945/2009(H1N1) | RV2945/2009 | HQ241051 | HQ240844 | HQ240637 | HQ239602 | HQ240223 | HQ240016 | HQ239809 | HQ240430 |
| 223 | A/Canada-BC/RV2946/2009(H1N1) | RV2946/2009 | HQ241052 | HQ240845 | HQ240638 | HQ239603 | HQ240224 | HQ240017 | HQ239810 | HQ240431 |
| 224 | A/Canada-BC/RV2947/2009(H1N1) | RV2947/2009 | HQ241053 | HQ240846 | HQ240639 | HQ239604 | HQ240225 | HQ240018 | HQ239811 | HQ240432 |
| 225 | A/Canada-BC/RV2948/2009(H1N1) | RV2948/2009 | HQ241054 | HQ240847 | HQ240640 | HQ239605 | HQ240226 | HQ240019 | HQ239812 | HQ240433 |
| 226 | A/Canada-BC/RV2949/2009(H1N1) | RV2949/2009 | HQ241055 | HQ240848 | HQ240641 | HQ239606 | HQ240227 | HQ240020 | HQ239813 | HQ240434 |
| 227 | A/Canada-BC/RV2950/2009(H1N1) | RV2950/2009 | HQ241056 | HQ240849 | HQ240642 | HQ239607 | HQ240228 | HQ240021 | HQ239814 | HQ240435 |
| 228 | A/Canada-ON/RV2961/2009(H1N1) | RV2961/2009 | HQ241057 | HQ240850 | HQ240643 | HQ239608 | HQ240229 | HQ240022 | HQ239815 | HQ240436 |
| 229 | A/Canada-ON/RV2962/2009(H1N1) | RV2962/2009 | HQ241058 | HQ240851 | HQ240644 | HQ239609 | HQ240230 | HQ240023 | HQ239816 | HQ240437 |
| 230 | A/Canada-ON/RV2963/2009(H1N1) | RV2963/2009 | HQ241059 | HQ240852 | HQ240645 | HQ239610 | HQ240231 | HQ240024 | HQ239817 | HQ240438 |
| 231 | A/Canada-ON/RV2964/2009(H1N1) | RV2964/2009 | HQ241060 | HQ240853 | HQ240646 | HQ239611 | HQ240232 | HQ240025 | HQ239818 | HQ240439 |
| 232 | A/Canada-ON/RV2965/2009(H1N1) | RV2965/2009 | HQ241061 | HQ240854 | HQ240647 | HQ239612 | HQ240233 | HQ240026 | HQ239819 | HQ240440 |
| 233 | A/Canada-ON/RV2966/2009(H1N1) | RV2966/2009 | HQ241062 | HQ240855 | HQ240648 | HQ239613 | HQ240234 | HQ240027 | HQ239820 | HQ240441 |
| 234 | A/Canada-ON/RV2967/2009(H1N1) | RV2967/2009 | HQ241063 | HQ240856 | HQ240649 | HQ239614 | HQ240235 | HQ240028 | HQ239821 | HQ240442 |
| 235 | A/Canada-ON/RV2983/2009(H1N1) | RV2983/2009 | HQ241064 | HQ240857 | HQ240650 | HQ239615 | HQ240236 | HQ240029 | HQ239822 | HQ240443 |
| 236 | A/Canada-ON/RV2984/2009(H1N1) | RV2984/2009 | HQ241065 | HQ240858 | HQ240651 | HQ239616 | HQ240237 | HQ240030 | HQ239823 | HQ240444 |
| 237 | A/Canada-ON/RV2985/2009(H1N1) | RV2985/2009 | HQ241066 | HQ240859 | HQ240652 | HQ239617 | HQ240238 | HQ240031 | HQ239824 | HQ240445 |
| 238 | A/Canada-NFL/RV3019/2009(H1N1) | RV3019/2009 | HQ241067 | HQ240860 | HQ240653 | HQ239618 | HQ240239 | HQ240032 | HQ239825 | HQ240446 |
| 239 | A/Canada-PQ/RV3189/2009(H1N1) | RV3189/2009 | HQ241068 | HQ240861 | HQ240654 | HQ239619 | HQ240240 | HQ240033 | HQ239826 | HQ240447 |
| 240 | A/Canada-MB/RV3607/2009(H1N1) | RV3607/2009 | HQ241069 | HQ240862 | HQ240655 | HQ239620 | HQ240241 | HQ240034 | HQ239827 | HQ240448 |
