# Supplementary material for: Nationwide Molecular Surveillance of Pandemic H1N1 Influenza A Virus Genomes: Canada, 2009
Source: PLoS One. 2011 Jan 7;6(1):e16087. doi: 10.1371/journal.pone.0016087 (PMC3017559; doi:10.1371/journal.pone.0016087)
Supplement: Table S9 — Accession numbers for the global outbreak A/H1N1pdm virus reference sequences used for clade assignment in Figure 3. (DOC) [file pone.0016087.s011.doc]

**Table S9**. Accession numbers for the global outbreak A/H1N1pdm virus reference sequences used for clade assignment in Figure 3.

| **Full Name** | **Accession Number** | **Segment Order** |
| --- | --- | --- |
| A/Bethesda/SP506/2009(H1N1) | ACU30100-9 | PB2 PB1 PA HA NP NA M1 M2 NS1 NS2 |
| A/Bogota/0466N/2009(H1N1) | ACU30070-9 | PB2 PB1 PA HA NP NA M1 M2 NS1 NS2 |
| A/Brawley/40081/2009(H1N1) | ACT79140-9 | PB2 PB1 PA HA NP NA M1 M2 NS1 NS2 |
| A/California/04/2009(H1N1) | ACP41102-11 | PB2 PB1 PA HA NP NA M1 M2 NS1 NS2 |
| A/California/06/2009(H1N1) | ACP52562-4 ACP41935-41 | NS1 NS2 NA HA NP M1 M2 PB2 PA PB1 |
| A/California/07/2009(H1N1) | ACP44170-6 ACP44183 ACP44189 ACQ63272 | M1 M2 NS1 NS2 PA PB2 PB1 NP HA NA |
| A/California/14/2009(H1N1) | ACQ76305-14 | NP PB1 PB2 NA PA NS2 NS1 M1 M2 HA |
| A/Cherry Point/WR0100/2009(H1N1) | ACY77580-9 | PB2 PB1 PA HA NP NA M1 M2 NS1 NS2 |
| A/Colorado/03/2009(H1N1) | ACQ76406-8 ACU13065-6 ACT36639-40 ACR38819 ACR38860 ACR19319 | NP NA HA M1 M2 NS2 NS1 PB2 PA PB1 |
| A/Craven/WR0019/2009(H1N1) | ACY77531-40 | PB2 PB1 PA HA NP NA M1 M2 NS1 NS2 |
| A/Denmark/523/2009(H1N1) | ACU00929-38 | PB2 PB1 PA HA NP NA M1 M2 NS1 NS2 |
| A/England/195/2009(H1N1) | ACR15612-21 | PA PB1 PB2 NS1 NS2 NP NA M1 M2 HA |
| A/Florida/04/2009(H1N1) | ACR08591 ACR08584 ACR08578 ACR08565 ACR08555-6 ACR08526 ACR38846 ACU13037-8 | NP PB1 PB2 NA M1 M2 HA PA NS1 NS2 |
| A/Houston/10OS/2009(H1N1) | ACZ96729-38 | HA M1 M2 NA NP NS1 NS2 PA PB1 PB2 |
| A/Italy/127/2009(H1N1) | ACT66152-61 | PB2 PB1 PA HA NP NA M1 M2 NS1 NS2 |
| A/Kansas/03/2009(H1N1) | ACQ76337-40 ACR19305 ACR19289 ACR19290 ACR38803 ACR38850 ACS72673 | NP NS1 NS2 HA PB1 M1 M2 PB2 PA NA |
| A/Korea/01/2009(H1N1) | ACQ84451-6 ACQ89891 ACQ08501-3 | HA NP M2 M1 NS2 NS1 NA PB2 PA PB1 |
| A/Malaysia/820/2009(H1N1) | ACY26189-98 | PB2 PB1 PA HA NP NA M1 M2 NS1 NS2 |
| A/Maryland/05/2009(H1N1) | ACR08598 ACR08587 ACR08580 ACR08514-5 ACR08538 ACS72686 ACS94470-1 ACR38856 | NP PB1 PB2 NS1 NS2 HA NA M1 M2 PA |
| A/Massachusetts/06/2009(H1N1) | ACQ76315-7 ACR19307 ACR19269-70 ACR19293-4 ACT36697 ACT36645 | NP NA HA PB1 NS2 NS1 M2 M1 PB2 PA |
| A/Mexico City/004/2009(H1N1) | ACY77974-83 | HA M1 M2 NA NP NS1 NS2 PA PB1 PB2 |
| A/Mexico City/WR1100N/2009(H1N1) | ACY77750-9 | PB2 PB1 PA HA NP NA M1 M2 NS1 NS2 |
| A/Mexico/4108/2009(H1N1) | ACQ99673 ACQ99660 ACQ99649-50 ACQ99635 ACQ99621 ACQ99605-6 ACT09108 ACR38785 | NP PA NS1 NS2 NA HA M1 M2 PB1 PB2 |
| A/Moscow/01/2009(H1N1) | ACR33740-3 ACR78464-9 | NA M2 M1 HA PB2 NP NS1 NS2 PA PB1 |
| A/Netherlands/602/2009(H1N1) | ACV84594-7 ACQ45339 ACV82598-601 ACQ45338 | PB2 PB1 PA NP NA M1 M2 NS1 NS2 HA |
| A/New Bern/WR0670/2009(H1N1) | ACY77660-9 | PB2 PB1 PA HA NP NA M1 M2 NS1 NS2 |
| A/New York/06/2009(H1N1) | ACQ63210-5 ACR38798 ACR38849 ACR19308 ACR19259 | M1 M2 NS1 NS2 NA NP PB2 PA PB1 HA |
| A/New York/3177/2009(H1N1) | ACS27189-98 | HA M1 M2 NA NP NS1 NS2 PA PB1 PB2 |
| A/New York/3324/2009(H1N1) | ACT85999-6008 | HA M1 M2 NA NP NS1 NS2 PA PB1 PB2 |
| A/New York/4735/2009(H1N1) | ACZ17100-9 | HA M1 M2 NA NP NS1 NS2 PA PB1 PB2 |
| A/Ohio/07/2009(H1N1) | ACQ63287-2 ACR08585 ACR08579 ACS72674 ACT36669 | M1 M2 NS2 NS1 PA HA PB1 PB2 NA NP |
| A/Rhode Island/02/2009(H1N1) | ACS94563 ACR67335-6 ACR67159 ACR67204 ACR67120 ACR67132-3 ACR67190 ACR67116 | PA M1 M2 NP NA PB1 NS1 NS2 HA PB2 |
| A/San Salvador/0169T/2009(H1N1) | ACY77620-9 | PB2 PB1 PA HA NP NA M1 M2 NS1 NS2 |
| A/Santo Domingo/0573N/2009(H1N1) | ACT22501-10 | PB2 PB1 PA HA NP NA M1 M2 NS1 NS2 |
| A/Singapore/ON305/2009(H1N1) | ACY46100-9 | PB2 PB1 PA HA NP NA M1 M2 NS1 NS2 |
| A/Taiwan/T1773/2009(H1N1) | ACU30006-15 | PB2 PB1 PA HA NP NA M1 M2 NS1 NS2 |
| A/Texas/42114261/2009(H1N1) | ACZ17488-97 | HA M1 M2 NA NP NS1 NS2 PA PB1 PB2 |
| A/Utah/05/2009(H1N1) | ACU13085 ACU13039-40 ACU13047-8 ACU13102 ACU13114 ACU13111A CU13077 ACU13090 | NP NS1 NS2 M1 M2 PA PB2 PB1 NA HA |
| A/Wisconsin/629-D0008/2009(H1N1) | ACZ16290-9 | HA M1 M2 NA NP NS1 NS2 PA PB1 PB2 |
| A/Wisconsin/629-D01147/2009(H1N1) | ACZ16420-9 | HA M1 M2 NA NP NS1 NS2 PA PB1 PB2 |
